# Reactivity of Olanzapine and Tricyclic Antidepressants on the Protective Effects of Trolox on Lipid Peroxidation Evaluated Using Fluorescence Anisotropy, Electron Paramagnetic Resonance Spectrometry, and Thermal Analysis

**DOI:** 10.1021/acschemneuro.4c00702

**Published:** 2025-01-17

**Authors:** Yusuke Horizumi, Reo Tanada, Yuya Kurosawa, Miwa Takatsuka, Tomohiro Tsuchida, Satoru Goto

**Affiliations:** Faculty of Pharmaceutical Sciences, Tokyo University of Science, 2641 Yamazaki, Noda, Chiba 278-8510, Japan

**Keywords:** multiacting receptor-target antipsychotics (MARTA), tricyclic antidepressant (TCA), thiobarbituric acid reactive substance (TBARS), electron paramagnetic resonance (EPR), fluorescence anisotropy, singular value decomposition (SVD)

## Abstract

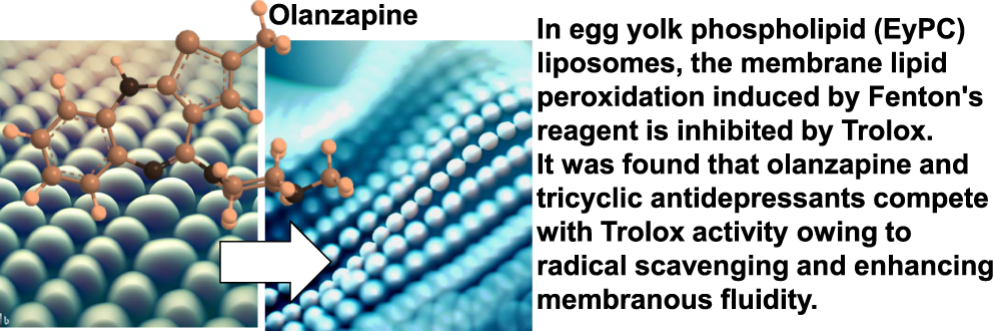

Multiacting receptor-targeting antipsychotics and tricyclic antidepressants stimulate various neurotransmitter receptors despite the different targets of postsynaptic receptors and presynaptic reuptake transporters. Their auxiliary and adverse effects may be caused by multiple targets or the modification of the neuronal membrane. To evaluate the membrane responses to olanzapine, imipramine, desipramine, amitriptyline, lidocaine, and dibucaine, we examined the inhibition of lipid peroxidation in egg yolk phosphatidylcholine liposomes. By contrast, their effects on membrane fluidity were measured as the suppressive contributions of the inhibitory activity of Trolox on lipid oxidation. These drugs inhibit lipid peroxidation and exclude harmful reactive oxygen species and the protective effect of Trolox. The fluorescence anisotropy of 1,6-diphenyl-1,3,5-hexatriene in saturated phospholipid liposome-containing drugs suggested that olanzapine, imipramine, and dibucaine enhanced membrane fluidity. The radical scavenging activity of 2,2-diphenylpicrylhidrazyl and galvinoxyl radicals was determined using electron paramagnetic resonance experiments, and their molecular flexibility was determined using thermograms for differential scanning calorimetry. Multiple regression analyses of the linear free energy relationship approach and comparative investigations revealed that the membranous fluidity of the liposomes, independent of the radical scavenging activity of the drugs, induced the inhibitory activity on lipid peroxidation. We discussed how these drugs act on nervous membranes and aimed to identify the relationship between uncertified functions and membranous fluidity.

## 1 Introduction

Multiacting receptor-targeting antipsychotics (MARTA) are atypical antipsychotics (AAPs, i.e., second-generation) that include serotonin–dopamine antagonists (SDAs, e.g., risperidone), dopamine system stabilizers, and serotonin–dopamine activity modulators.^1−3^ Conventional typical antipsychotics (TAPs, i.e., first-generation antipsychotics such as haloperidol) dominantly sedate the excitatory nervous system with dopaminergic function. MARTA and SDA improve this by adding an inhibitory effect to the repressive nervous system via serotoninergic function.^4,5^ Generally, the mechanisms of AAPs remain unclear, and a “black box” warning has been issued^5−10^ MARTA, a term encompassing olanzapine (OLZ), clozapine (CLZ), quetiapine (QTP), and asenapine, is a promising approach for the treatment of schizophrenia, bipolar disorder, autism, irritability, and depression. It stands out from other AAPs because of its unique ability to inhibit or stimulate dopamine D2 and serotonin 5HT2A receptors and a diverse combination of various receptor subtypes.^11^

This study has significant implications for the pharmaceutical understanding of MARTA, particularly OLZ. The authors aimed to verify its potential to significantly reduce adverse effects on the functional regulation of repressive and excitatory nerves. OLZ binds specifically to lipid membranes, suggesting that a direct, nonspecific effect on cell membranes may be crucial for its activity.^12,13^ Despite its reputation for reducing the extrapyramidal symptoms characteristic of schizophrenia via the antagonism of D2 receptors,^13^ specific binding of OLZ to lipid membranes^14,15^ implies that the antagonism of various practical receptors might indirectly weaken them through direct action on cell membranes.^16^

OLZ experimentally inhibits the subtype-specific ligand binding to dopamines D1, D2, and D4; serotonins 5HT2A, 5HT2C, and 5HT3; α1-adrenergic; histamine H1; and postsynaptic muscarinic M1 receptors.^17^ Although CLZ is comparable to OLZ, its affinity to the α1-adrenergic receptors is 18 times higher than that of OLZ, which might be related to the high adverse effect of CLZ.^17^ For SDAs, risperidone is specific to dopamines D2 and D4, especially serotonin 5HT2A and α-adrenergic receptors, but not to muscarinic and histamine receptors. Haloperidol is not tolerated by TAPs, except for dopamine receptors. However, agonistic probe adsorption assays might not demonstrate that binding is competitive; therefore, evidence of MARTA antagonists binding to multiple agonist-specific sites might be sufficient.

In the Protein Databank (PDB) of the Research Collaboratory for Structural Bioinformatics (RCSB), X-ray diffraction crystallographic structures of the haloperidol–D2 receptor complex, risperidone–D2 receptor complex (PDB entry, 6 cm4), and risperidone–5HT2A receptor complex (6a93) are published. However, those for MARTAs can only be identified for the CLZ–histamine H4 receptor complex (8jxv). Meanwhile, OLZ and CLZ induce rapid weight gain by influencing insulin levels. Zuclopenthixol, an SDA antipsychotic, and haloperidol cannot increase blood insulin compared with OLZ and CLZ.^18^ This demonstrates the selectivity of OLZ and CLZ in stimulating the peripheral site to improve insulin levels.

Cytochrome P450 (CYP) enzymes generally have broad selectivity for reactive drugs with multiple affinities to their subtypes. OLZ is recognized mainly by CYP of family 1-subfamily A2 (CYP1A2) and partially by CYP2D6 as a substrate, whereas CLZ has an affinity for CYP1A2 with an additional contribution from CYP3A4. CYP3A4 exclusively metabolizes QTP and ziprasidone, and risperidone shows selectivity for CYP2D6 and, to a lesser extent, CYP3A4.^19^ CYP1A2, CYP2D6, and CYP3A4 selectively distinguish OLZ and CLZ from others, risperidone and weakly OLZ from others, and QTP and partially CLZ and risperidone from OLZ, respectively. MARTA and SDA maintain decisive specificity for CYP metabolism. However, the inhibitory effects of AAPs for CYPs are inconsistent with the CYP substrate-specificity spectrum.^19−21^ Molecular recognition of the binding site for substrates and inhibitors (as well as agonists and antagonists) may occur without generality. Herein, the molecular recognition of example proteins is presented, but not their metabolism, delivery, distribution, or excretion.

Furthermore, broad selectivity for pharmaceuticals and chemicals is generally observed in serum albumins.^22−24^ Their intrinsic fluorescence from mainly tryptophan residues localized at the drug binding site was observed, with OLZ binding dose-dependently. For bovine serum albumin containing two tryptophan residues, endothermic loose binding was encouraged at increased temperatures, suggesting that faster diffusion of the ligand enhanced collisions, thus increasing quenching (dynamic quenching).^22^ For human serum albumin, which contains a single tryptophan residue, the apparent binding constant of OLZ to albumin decreases with increasing temperature.^23^ Thus, the thermal fluctuation of the exothermal tight binding between the ligand and tryptophan residue attenuated the residual fluorescence (static quenching). OLZ demonstrated a more selective interaction with the binding site of human serum albumin than with the bovine one.

Warfarin, a site-specific marker, competes with OLZ for protein binding.^24^ This competition was expected, considering the proximity of the tryptophan residue to the warfarin-specific site. Another drug-binding site of human serum albumin binds diazepam, a 1,4-benzodiazepine derivative (BDZ). The molecular interaction of diazepam with human serum albumin was elucidated in the crystallographic structure of the diazepam–human serum albumin complex (2bxf). Despite the similarity of the thienobenzodiazepine backbones of OLZ and diazepam, OLZ was rarely inhibited by a diazepam-specific site marker, confirming its high site selectivity to serum albumins.^25−27^ However, no evidence of OLZ tolerance was observed for these proteins.

BDZs are antidepressants with sedative, hypnotic, anxiolytic, anticonvulsant, and muscle relaxant effects. They enhance the stimulation of the inhibitory neurotransmitter γ-aminobutyric acid (GABA) via GABA-A receptors. Chuang and Otagiri have proposed high selectivity of human serum albumin diazepam-specific sites from comparative experiments for diazepam, clonazepam, and other BDZs.^27,28^ Meanwhile, the crystallographic structures of BDZ-GABA-A receptors (6 × 3x and 6hup) show that BDZs are localized in both the soluble loop region and transmembrane domains. Similar to diazepam, flumazenil, a competitive benzodiazepine antagonist without a stimulating effect, binds to a particular site at the interface between its subunits in the loop region (6 × 3u, 6d6t, and 6d6u).^29^ However, the binding sites of general anesthetics (etomidate, propofol, and phenobarbital) as allosteric ligands are located in the transmembrane domain of GABA-A receptors.^30^

Clinical surgery requires general anesthesia induction to avoid severe pain. In 1802, Seishu̅ Hanaoka used a botanic combination medicine, and in 1864, William Morton used ether inhalation; such discoveries have benefitted the human population for over 200 years.^31−33^ Although Meyers and Overton certified 100 years ago that the hydrophobicity of various volatile chemicals is an essential indicator that correlates with their anesthetic activity,^34,35^ details of the anesthetic mechanism remain unclear.^36,37^ Peterson et al. verified that isoflurane and chloroform perturb lipid rafts and fluid lipid regions composed of sphingolipids and cholesterol, demonstrating that ganglioside rafts and bulk lipids are thoroughly mixed.^37^ These inhalation anesthetics disrupt lipid rafts, and their effect on lipids leads to phospholipase D2 activation. This inspired us to reevaluate the impact of lipid membrane-acting drugs on biomembranes. Similar possibilities have been reported for general anesthetics, local anesthetics (LAs), and neurotropics, which may act on central nervous membranes.^38^ Banrjee’s group proposed that phospholipases facilitate the catabolism of lipid membranes.^39,40^

The unique properties of LAs and neurotropics have attracted research attention. These drugs exert their pharmacological effects by interacting with biomembranous proteins.^41,42^ Neurotropics, including antipsychotics and antidepressants, are particularly intriguing because of the unclear causal relationship between functional protein modifications and their impact on neurological function.^43,44^ One reason for these direct interactions with biomembranes is that LAs and neurotropics are amphiphilic cations. This unique characteristic allows them to interact electrostatically with the negatively charged moieties on the membrane surface.^45,46^ Another reason is the high hydrophobicity of LAs and neurotropics. They penetrate biomembranes to modify their physical properties, thereby increasing biomembrane fluidity.^47^ LAs inhibit chemical transmission in neurons,^48^ thus demonstrating a correlation between their effect on the cardiolipin-containing biomembrane with enhanced cardiotoxicity^49^ and disruption of lipid rafts.^50^

Horizumi et al. have reported that LAs, lidocaine (LDC), and dibucaine (DBC) inhibit membrane-protective functions.^51^ These assessments can be used to elucidate how the direct effects of drugs on biomembranes affect their pharmacological functions and side effects.^51^ The fine structure of biomembranes makes it difficult to investigate lipid raft stability with drugs. Liposomes, which are models consisting of purified lipids of biomembranes, are widely used to reveal interactions between lipid membranes and medicines. Thus, Horizumi’s assessment method using egg yolk lecithin (EyPC) liposomes mimics the biomembranes abundant in polyunsaturated fatty acids (PUFAs), such as linolates, linolenates, and arachidonates.^52^

Lipid peroxidation is an irreversible chain reaction in EyPC liposomal PUFAs that is caused by oxidative stress.^53,54^ Oxidative stress causes partial brain atrophy through lipid peroxidation.^55^ Such brain atrophy can lead to depression and schizophrenia.^55,56^ The membrane-protective function was examined by inhibiting the antioxidative activity of Trolox (TRO, 6-hydroxy-2,5,7,8-tetramethylchroman-2-carboxylic acid). Takara et al. verified that DBC and LDC could not scavenge radicals but protected ketoprofen from degradation induced by ultraviolet irradiation, proving that LAs are inactive in radical reactions.^57^ Thus, LAs that reduce lipid membrane fluidity indirectly influence the antioxidant activity of TRO, according to Horizumi’s assessment method.^51^

The present study used this method to investigate the comparative effects of LAs and OLZ and examined the impact of antihistaminic diphenhydramine (DHM) and tricyclic antidepressants (TCAs).

## 2 Results

### 2.1 Lipid Peroxidation of EyPC Liposomal PUFAs in Small Unilamellar Vesicles (SUVs) Induced by Fenton’s Reagent

Our experimental setup was meticulously designed to produce hydroxyl radicals, the reactive oxygen species, using Fenton’s reagents, i.e., Fe(NH_4_)_2_(SO_4_)_2_ at a final concentration of 0.2 mM and H_2_O_2_ of 0.1 mM. These radicals induce the lipid peroxidation of PUFAs in EyPC liposomes, which form SUVs with a log-average diameter of 61.0 nm measured by dynamic light scattering (DLS). The organic phosphorus concentration in the liposomal suspension was adjusted to 1.3 μg/μL. Termination products such as malondialdehyde (MDA) and other byproducts were obtained from radical chain reactions. Their chromogenic reaction with thiobarbituric acid (TBA) produces a TBA-reacting substance (TBARS), which is calibrated with the standard TBARS generated from the TBA reaction of tetraethoxypropane as an MDA source.

Figure S1A shows the TBARS spectra produced by the chromogenic reaction with various concentrations of MDA. These spectra, which expanded proportionally with MDA concentration, contained a peak at 530 nm and a shoulder at 495 nm. Figure S1B further illustrates the lipid peroxidation of SUVs, which presents TBARS spectra with bimodal peaks at 455 and 530 nm. These peaks were magnified to correspond to their organic phosphorus concentration. In general, a calibration line was produced using the observed absorbance at a wavelength of approximately 530 nm.

To demonstrate the nature of the 455 nm signal, Figure S1C shows the TBARS spectra from a mixture of various concentrations of TRO and Fenton’s reagent without lipids. We discovered a 455 nm signal, indicating that this signal is a nonspecific product generated by TBA chromogenic reactions. Figure S1C also reveals a co-occurrence between the 455 and 530 nm signal heights, suggesting the need for a purified intensity of the 530 nm signal corresponding to the standard MDA spectral peak. Therefore, applying a computation of a singular value decomposition (SVD) to distinguish the specific signal is recommended for analyzing the spectra, which is a crucial step in our research process.^27,51,58−69^

### 2.2 Competition of Imipramine (IMP) and Related Compounds with TRO for Antioxidative Activity in SUVs

Our previous study has indicated that LAs enhanced apparent 1-octanol/water partition coefficients, log *P*_obs_, of hydrophobic analgesic indomethacin, depending on the hydrophobicity and structural fluctuation of LAs evaluated by Yalkowski’s descriptors log *f* or thermodynamically measured fusion entropy Δ_fus_*S*^0^.^65^ Simultaneously, the apparent aqueous solubility of i ndomethacin was regulated by the additive LAs, correlating to hydrophobicity of LAs and the differential melting temperature Δ*T*_m_ owing to the mixing of the LAs.^69^ The antidepressant IMP, which contains log *P* = 4.80,^70^ greater than DBC’s log *P* = 4.40,^70^ resulted in a comparable attenuation of indomethacin’s log *P*_obs_. Quantitative structure–activity relationships (QSAR) interpreted its reason as the log *f* and Δ_fus_*S*^0^ of DBC were higher than IMP, certifying that the wealthy structural fluctuation of DBC with two side chains is adequate. Based on this achievement, protecting ketoprofen from ultraviolet irradiation photodegradation was successful because DBC entangled the target molecule.^57^

In the present study, we examined IMP and its related psychotropic drugs for functional modifications of liposomal membranes, comparing them to our previous observations of LDC and DBC.^51^ IMP is classified as a TCA, of which desipramine (DSP) is an example of the dibenzoazepine derivative and amitriptyline (AMT) as that of the dibenzocycloheptenylidene derivative were appended to the current examinations. These TCAs pharmacologically inhibit the presynaptic reuptake of noradrenaline and serotonin, which are neurotransmitters associated with depression.^71,72^ The levels and activities of reactive oxygen and nitrogen species modulate noradrenaline, serotonin, dopamine, and glutamate levels.^71,72^ Thus, oxidative stress may be relevant to the pathogenic mechanisms underlying depression and many major psychiatric disorders.^71,72^ Different TCA family members induce cardiotoxic oxidative stress.^73^ Moreover, TCAs might stimulate histaminergic H1, muscarinic, and presynaptic α1-adrenergic receptors. Based on this analogy, we adopted OLZ of the MARTA drug as a test sample.

**Figure 1:**
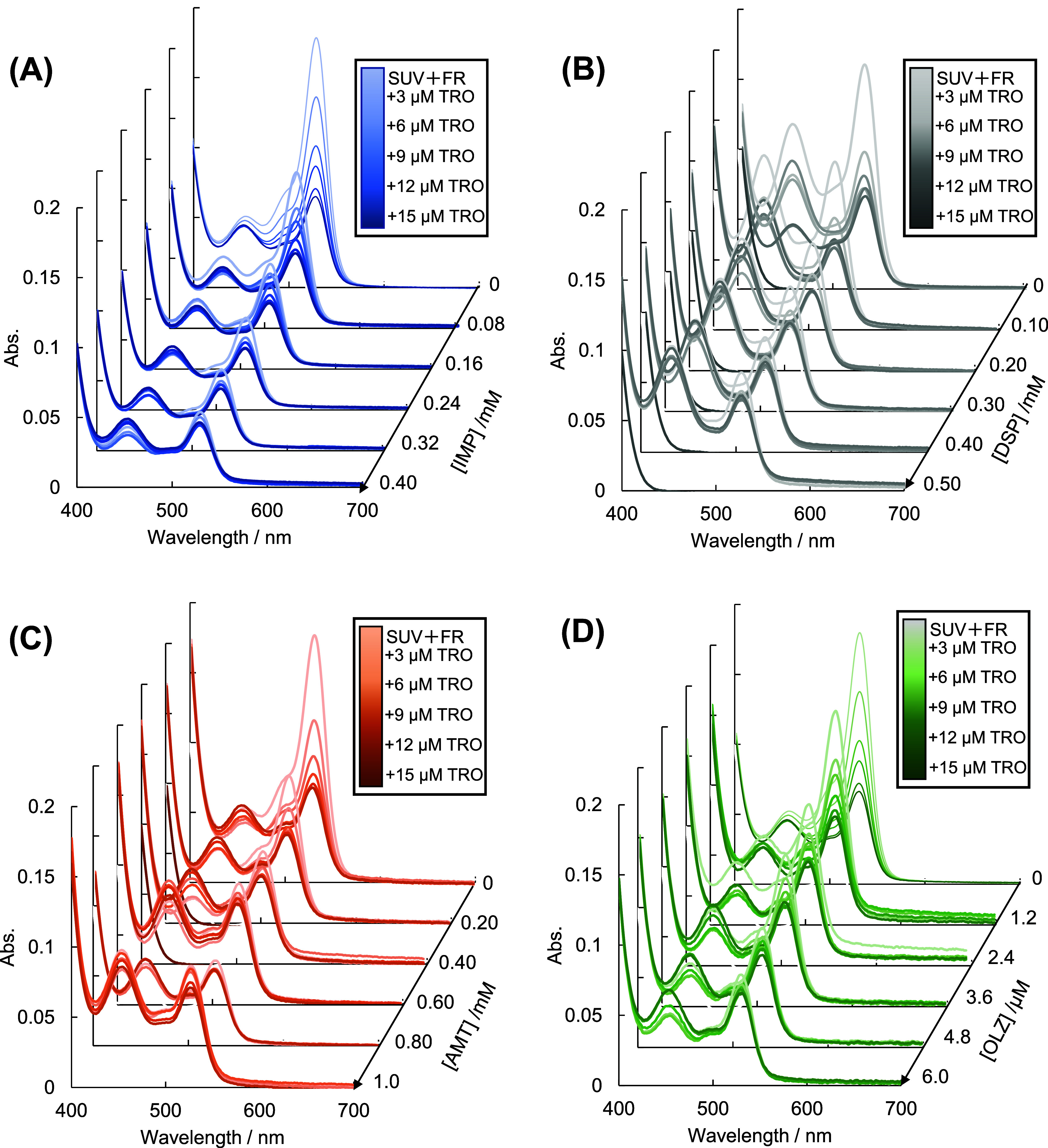
Observed spectra of thiobarbituric acid-reacting substance for lipid peroxidation inhibited by various concentrations of 6-hydroxy-2,5,7,8-tetramethylchroman-2-carboxylic acid with 0–0.40 mM imipramine (A), 0–0.5 mM desipramine (B), 0–1.0 mM, amitriptyline (C), and 0–6.0 μM olanzapine (D). Lipid peroxidation in liposomes (phosphorus concentration of 1.3 μg/μL) was induced by adding Fenton’s reagent at 310 K in a phosphate buffered saline–ethanol mixture at 9:1 for 12 min. After the appropriate period, adding 2% w/v butylated hydroxytoluene terminated lipid peroxidation.

Figure 1A shows the spectral attenuation of TBARS, which was generated by the lipid peroxidation of EyPC liposomal PUFAs, depending on the concentration of TRO. As the TRO concentration increased, the 530 nm signal intensity gradually decreased, and the 455 nm signal intensity slightly declined. The 530 nm signal corresponded to lipid peroxidation products, and its decrements reflect the function of TRO in protecting against peroxidative membrane disruption. The TBARS spectral changes depended on the TRO concentration at IMP concentrations of 0.08, 0.16, 0.24, 0.32, and 0.40 mM. With or without TRO, IMP inhibited lipid peroxidation. The coexistence of TRO and IMP independently inhibited lipid

In our previous report,^51^ LDC (log *P* = 2.26)^70^ and DBC (log *P* = 4.40) inhibited lipid peroxidation in a hydrophobic manner. For the present experiments, the hydrophobicity of the psychotropic drugs was surveyed as OLZ (log *P* = 4.094), IMP (4.80), DSP (4.90), and AMT (4.92).^70,74−76^ The preliminary screening revealed that the 530 nm signals overlapped onto the lowest intensity spectra (i.e., 100% inhibition) without TRO, which was observed at the lowest concentrations of 0.4 mM for IMP, 0.5 mM for DSP, 1.0 mM for AMT, and 6.0 μM for OLZ. According to a previous study,^51^ they were 3.2 mM for LDC and 2.0 mM for DBC. Drawing a rational causality between hydrophobicity and the lowest concentration would be difficult. OLZ, which has relatively poor hydrophobicity, accessed the deep regions of the lipid bilayer and inhibited lipid peroxidation at significantly lower concentrations than IMP, DSP, AMT, and DBC. The latent ability of OLZ at a very low concentration significantly contradicted our previous interpretation of its contribution to the apolar intermolecular interaction, which comprised cardinal hydrophobicity and structural fluctuation as auxiliary factors.

### 2.3 Effects of Psychotropic Drugs and TRO on Lipid Peroxidation

SVD computation, which is a precise and reliable method, was applied to extract the legitimate spectral components of TBARS derived from lipid peroxidation.^51^ The SVD processing extracts the average spectra as the first component and the differential spectra to reproduce the spectrum under specific experimental conditions. Therefore, the linear combination of coefficients multiplied by the basic functions for the required components yields the reproductions. The third or subsequent components are compensated if the first and second components are insufficient for reproduction.^58−67^ The analyzed matrix consisted of the observed spectra (Figures 1 and S1), and the Supporting Information as vertical vectors to be lined up in the lateral direction. The SVD computation for this analyzed matrix was divided into the basis function matrix, diagonal matrix of singular values, and matrix of singular vectors as coefficients for the linear combinations to reproduce the individual spectra from left to right. The number of components required corresponded to the cumulative variance presented along with the plots of singular values in Figure S2A. As those of the first and second components and the top three components were 85.1% and 90.9%, the reproducibility of the linear combinations would be sufficient.

As described above, the basis function ψ_1_ of the first component was presumed to correspond to the average. Figure S2B demonstrates that the calculated basis spectrum ψ_1_ (blue) containing the 530 nm signal would be appropriate for an average of all spectra in Figures 1 and S1. The second component’s basis function ψ_2_ (amber) indicated the incremental 530 nm signal and decremental 455 nm signal. This would act to dissolve the nonquantitative co-occurrence of the 455 nm signal. The basis function ψ_3_ (gray) would be a parallel contribution between the 455 and 530 nm signals, although it may be ineffective.

Figure S3 shows the singular vectors of the first, second, and third components gathered in the spectra at various concentrations of TRO with and without IMP (Figure S3A–C), AMT (Figure S3D–F), DSP (Figure S3G–I), and OLZ (Figure S3J–L). The coefficients λ_1_ of the first component are invariable to TRO concentration as the average contribution of the first basis function. The second coefficient λ_2_ attenuated depending on the concentration of psychotropic drugs. This suggests that the second component reflects the intensity of the 530 nm signal and cancels the co-occurrence of the 455 nm signal. The third coefficient λ_3_ irregularly fluctuated, and the corresponding singular value σ_3_ was small (Figure S2A). Hence, the first and second components with ω = −σ_1_λ_1_+σ_2_λ_2_ were adopted for later representation.

**Figure 2:**
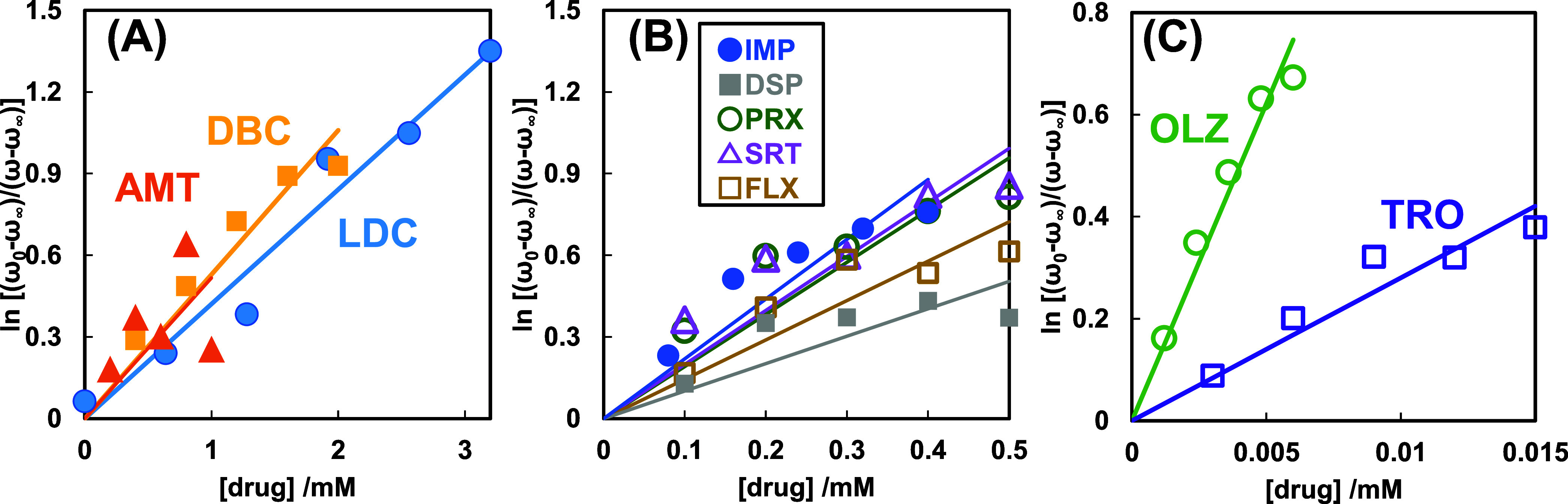
Diagrams between the negative logarithm of the SVD component score change (ω–ω_∞_) relative to the maximum range (ω_0_–ω_∞_) and the concentrations of LDC, DBC, AMT (A), IMP, DSP, PRX, SRT, FLX (B), OLZ, and TRO (C). The score ω is the linear combination of the singular vectors multiplied by the singular values in the first and second components derived from the SVD analysis for the observed spectra (Figure 1) containing calibration spectra (Figure S1). As the intensity of the ordinates indicates the inhibitory effect of drugs or TRO on lipid peroxidation, the concentration (abscissa) at ln 2 = 0.693 on the ordinate was defined as the IC_50_ of drugs or TRO. Although the antidepressants demonstrate upward convex curves, the inhibitory activity for convenience was evaluated as the gradient of the regression line. These drugs require further investigation.

The negative logarithm of the SVD component score change (ω–ω_∞_) relative to the maximum range (ω_0_–ω_∞_) was plotted as the ordinate, in which the inhibitory effects can be inversely proportional to the purified intensity of TBARS. Figure 2 demonstrates that the psychotropic drug concentration inhibited the lipid peroxidation of EyPC liposomal PUFAs in SUVs. Similar to a previous report,^51^ the gradient of hydrophobic DBC, which indicated an inhibitory effect, was slightly more significant than that of LDC. Although the hydrophobicity of AMT was 0.52, which was higher in the logarithmic unit of log *P* (i.e., three times in *P*) than that of DBC, AMT was comparable with DBC in the evaluated inhibitory effect (Figure 2A). Figure 2B shows that the dibenzoazepine derivatives IMP and DSP showed large slopes at concentrations of <0.2 mM, although they did not stretch at higher concentrations. Paroxetine (PRX), sertraline (SRT), and fluvoxamine (FLX) provided mediocre upward convex curves (Figure 2A); therefore, conventional and subsequent antidepressants would require comprehensive investigation. The inhibitory potency of OLZ was 3–4 times higher than that of TRO.

### 2.4 Quantitative Evaluation of Drug Effects as Determined by the Inhibitory Activity of TROs on Lipid Peroxidation

Figure S4A–D shows the regression lines for the inhibition of lipid peroxidation by psychotropic drugs with TRO at various low concentrations. Their slopes showed an inhibitory effect on psychotropic drugs, which weakened depending on the TRO concentration. Since TRO maintains a potent protective effect against the oxidant of Fenton’s reagent, which causes lipid peroxidation, the apparent antioxidant activity of the psychotropic drug may become invisible.

Conversely, Figure S4E–H shows the regression lines for the inhibition of lipid peroxidation by TRO with psychotropic drugs. Assuming that the TRO’s power hides the psychotropic drug’s effects, the TRO’s slope should be independent of the drugs. However, the antioxidant activity of TRO decreased concentration-dependently. We obtained the p*I*_50_ values converted from the obtained slopes because the ordinate consisted of relative figures.

**Figure 3:**
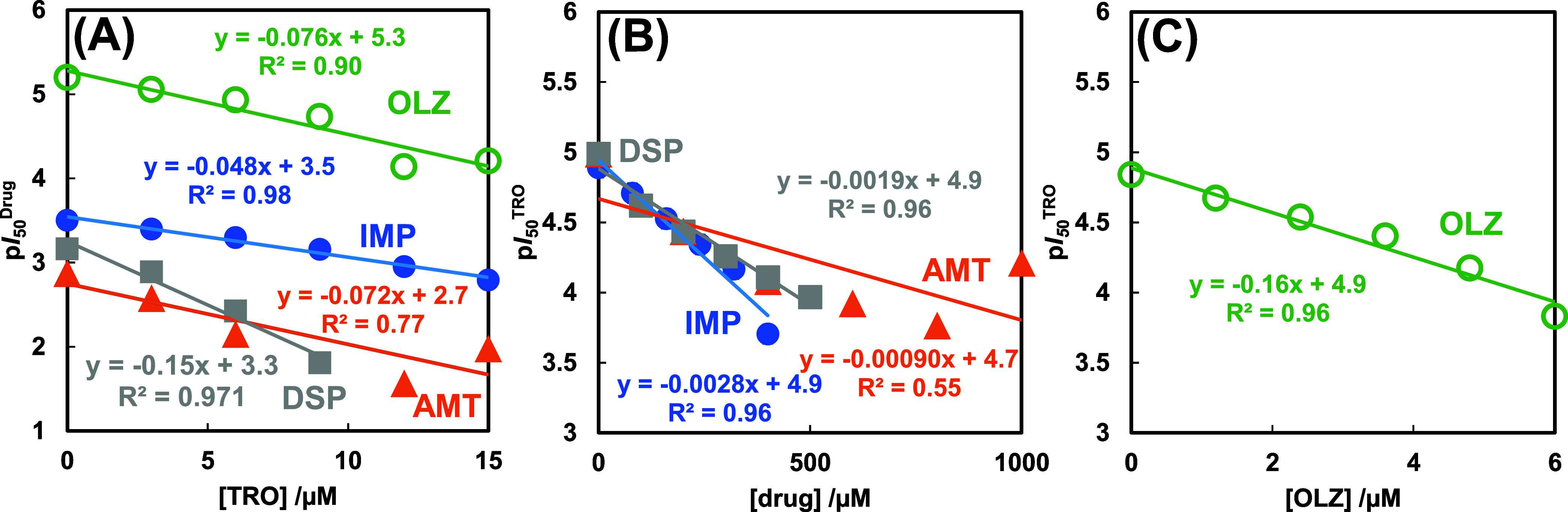
Diagrams between the p*I*_50_^Drug^ and TRO concentration (A) and between p*I*_50_^TRO^ and drugs’ concentration (B)/(C). In our previous study,^49^ regression lines of LDC and DBC were p*I*_50_^LDC^ = −0.041[TRO]+2.6 and p*I*_50_^DBC^ = −0.043[TRO]+2.8, respectively. Their effects on the TRO’s activity were p*I*_50_^TRO^ = −0.00021[LDC]+4.9 and p*I*_50_^TRO^ = −0.00042[DBC]+4.9, indicating the slope 1/10 less than the antidepressants.

Figure 3A shows the concentration-dependent effects of TRO on the inhibitory effects of psychotropic drugs on lipid peroxidation. The ordinate represents the drug’s p*I*_50_ in inhibiting lipid peroxidation, demonstrating the concentration dependence of the TRO concentration on the abscissa. AMT resulted in a regression line resembling that of DSP, although we could not represent quantitative results for AMT at high concentrations. In a previous study, although LAs did not act as antioxidants,^57^ they inhibited lipid peroxidation.^51^ The LAs enhanced liposome’s membranous barrier function, protecting them against hydroxyl radicals. We considered this function to be independent and to coexist with the scavenging of hydroxyl radicals by TRO. Thus, we considered a similar interpretation of psychotropic drugs.

Figure 3B,C show the intricate details of p*I*_50_^TRO^ scavenging of hydroxyl radicals, which also depends on the concentrations of IMP, DSP, AMT, and OLZ. The slopes of these figures indicate that TRO attenuated the inhibitory effect of each drug and are expressed as dp*I*_50_^Drug^/d[TRO] (Table 1). This complex interaction may be attributed to the scavenging of hydroxyl radicals by TRO.^51^ The elimination of hydroxyl radicals in the liposomal membranes could have prevented lipid peroxidation and reduced the p*I*_50_^Drug^. TCAs reduced TRO membrane protection at lower concentrations than DBC, despite being as hydrophobic as DBC. By contrast, OLZ reduced TRO activity at lower concentrations. This intricate balance of factors requires further research.

**Table 1:** p*I*_50_ Values on the Examinations of TBARS on Liposomal Lipid Peroxidation, DPPH, and GLV Radical Scavenging

Drug	TBARS p*I*_50_^Drug^	d[p*I*_50_^Drug^]/d[TRO]	TBARS p*I*_50_^TRO^	d[p*I*_50_^TRO^]/d[TRO]	DPPH p*I*_50_^Drug^	DPPH p*I*_50_^TRO^	GLV p*I*_50_^Drug^	GLV p*I*_50_^TRO^
none	-	-	4.9	-	-	4.0	-	4.1
LDC	2.5	–19. × 10^–3^	4.1	–0.21 × 10^–3^	<1.8	4.0	1.9	4.2
DBC	2.8	–21. × 10^–3^	4.2	–0.42 × 10^–3^	<2.0	4.0	0.33	4.2
DHM	2.7	-	4.6	-	-	-	-	-
IMP	3.7	–48. × 10^–3^	3.7	–2.8 × 10^–3^	2.4	4.0	2.4	4.2
AMT	3.3	–72. × 10^–3^	4.2	–0.90 × 10^–3^	2.1	4.0	2.4	4.3
DSP	2.7	–150. × 10^–3^	4.0	–1.9 × 10^–3^	2.2	3.9	1.4	4.3
OLZ	5.3	–48. × 10^–3^	3.4	–160. × 10^–3^	4.2	4.0	4.1	4.3
SRT	3.3	-	-	-	-	-	-	-
FLX	3.4	-	-	-	-	-	-	-
PRX	3.4	-	-	-	-	-	-	-

### 2.5 EPR Study for 2-Diphenylpicrylhidrazyl (DPPH) Radical Scavenging Activity of the Drugs

IMP, DSP, AMT, and OLZ showed an inhibitory effect on the membrane lipid peroxidation of EyPC liposomes in the absence of TRO. These drugs may act directly as antioxidants against lipid peroxidation. This section examines the radical scavenging activity of the drugs in ethanolic aqueous solvents and 1-octanol and their influence on the activity of TRO.

DPPH is a stable free radical dye that produces an intense violet color upon absorption at a wavelength of 517 nm. Since DPPH is radical-scavenged by antioxidants and converted to a yellow pigment, its colorimetric measurements have historically been used to evaluate the radical scavenging activity of antioxidants. The EPR signal pattern of DPPH, obtained as five almost equidistant peaks (EPR spectra are presented as differential curves), primarily corresponded to two equivalent nitrogen nuclei. The observed hyperfine coupling constants were *a*_N1_ = *a*_N2_ = 8.86 G (1 G = 0.1 mT).^60^

**Figure 4:**
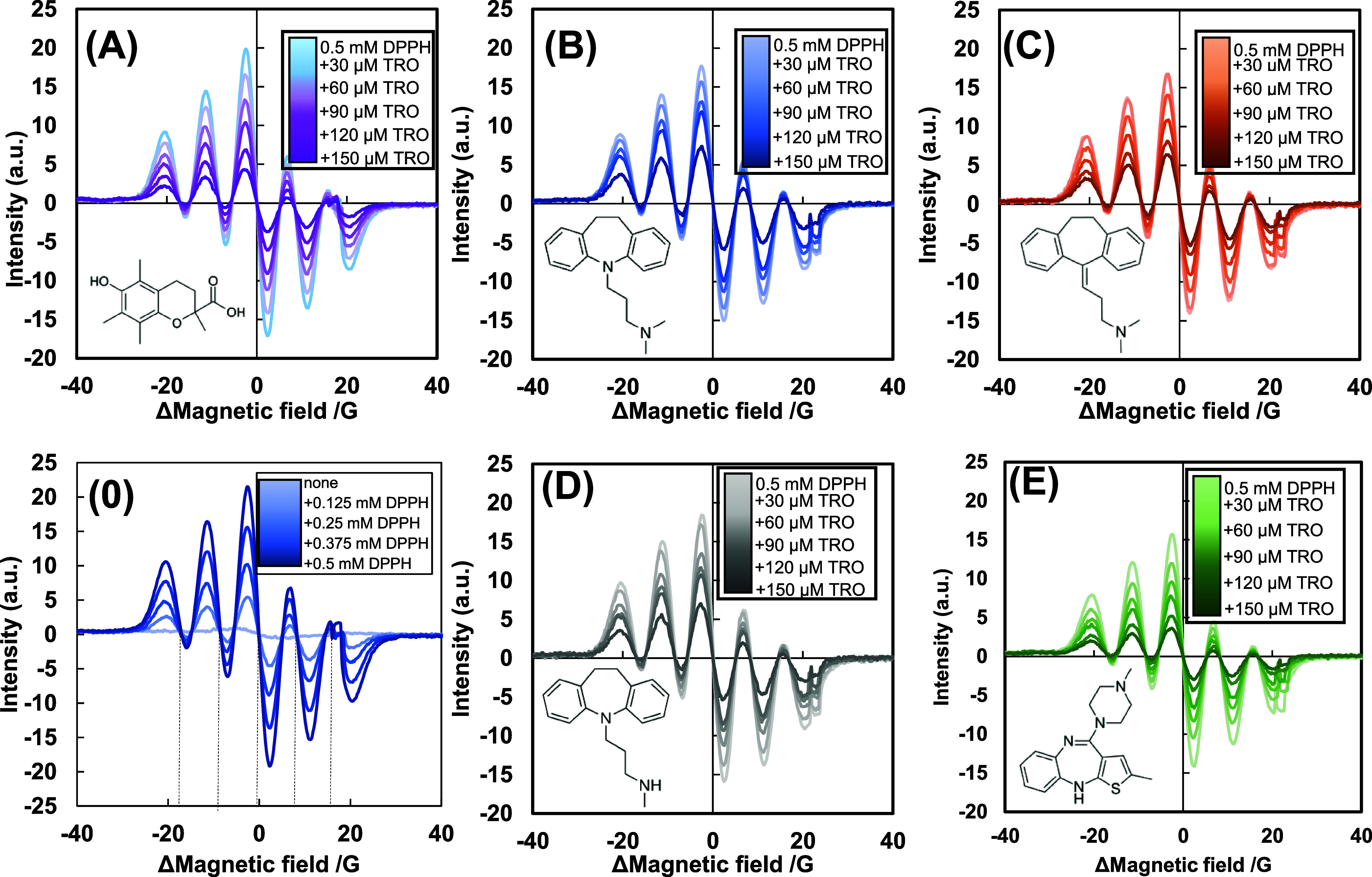
Electron spin resonance spectra of 0.5 mM 2-diphenylpicrylhidrazyl (DPPH) radicals inhibited by 0–150 μM TRO without (A) or with 2 mM imipramine (B), 5 mM amitriptyline (C), 2.5 mM desipramine; (D), and 30 μM olanzapine (E) for 30 min in 71% (v/v) ethanol/water. The abscissa was aligned to the individual rotatory symmetry point, indicating the magnetic field difference. Hyperfine coupling constants were derived from the abscissa intervals at observed invariable points (peaks of the integrated curve), independently of 0–0.5 mM DPPH concentrations on the decrement curve regions in (0), determined as a_N1_ = a_N2_ = 8.86 G corresponding to two equivalent nitrogen nuclei.

Figure 4 precisely illustrates the EPR spectra of the DPPH radicals in 71% (v/v) ethanol/water decayed with TRO. The abscissa is presented as the difference from the antisymmetry center to adjust the spectral phase in the SVD procedure. The signal intensity at a magnetic field difference of approximately −2.5 G (or ordinate distance between the highest peak at −2.5 G and the lowest trough at +2.5 G) decreased linearly with TRO concentration. Figure 4B–E further demonstrate the EPR spectra of DPPH radicals, showing their decay depending on the TRO concentrations of 2 mM IMP, 5 mM AMT, 2,5 mM DSP, or 30 μM OLZ, respectively. IMP, AMT, and DSP increased the −2.5 G signal intensity at the 150 μM TRO, indicating either their inhibition of TRO’s radical scavenging activity to DPPH radicals or their reproduction of DPPH radicals. Meanwhile, OLZ decreased this signal intensity, suggesting that OLZ either enhanced TRO’s activity or that scavenged DPPH radicals independently of TRO.

The EPR spectra of 0.5 mM DPPH radicals decayed depending on the drug concentration (Figure S5). LDC, DBC, IMP, AMT, and DSP slightly reduced the −2.5 G signals, whereas OLZ attenuated the −2.5 G signal intensity. To avoid inaccuracy caused by the choice of measured signals by chance, SVD analysis of the whole spectral shape was applied to the EPR spectra of the DPPH radicals (Figures 4 and S7). The obtained SVD parameters are summarized in Figure S6, which suggests that the first component is sufficient.^60^ A deviation from the latent antisymmetry of the EPR signal pattern may cause the third component.

Figure S7 shows the TRO scavenging of DPPH radicals in the absence and presence of drugs and their own activities, depending on the concentration. In Figure S7A, the absolute values of the slopes of the TRO scavenging DPPH radicals decreased with the drugs. The intercepts indicate specific plots on the lines in Figure S7B,C, reflecting the independent contributions of the drugs to TRO. The scavenging activities of IMP, AMT, and DSP on DPPH radicals were relatively lower than those of TRO, whereas that of OLZ was similar to that of TRO. Meanwhile, TRO activity (slope of −2.6 × 10^–3^) was significantly attenuated by IMP (−1.8 × 10^–3^), AMT (−1.9 × 10^–3^), DSP (−1.8 × 10^–3^), and OLZ (−1.9 × 10^–3^).

### 2.6 EPR Study for the Galvinoxyl (GLV) Radical Scavenging Activity of the Drugs

**Figure 5:**
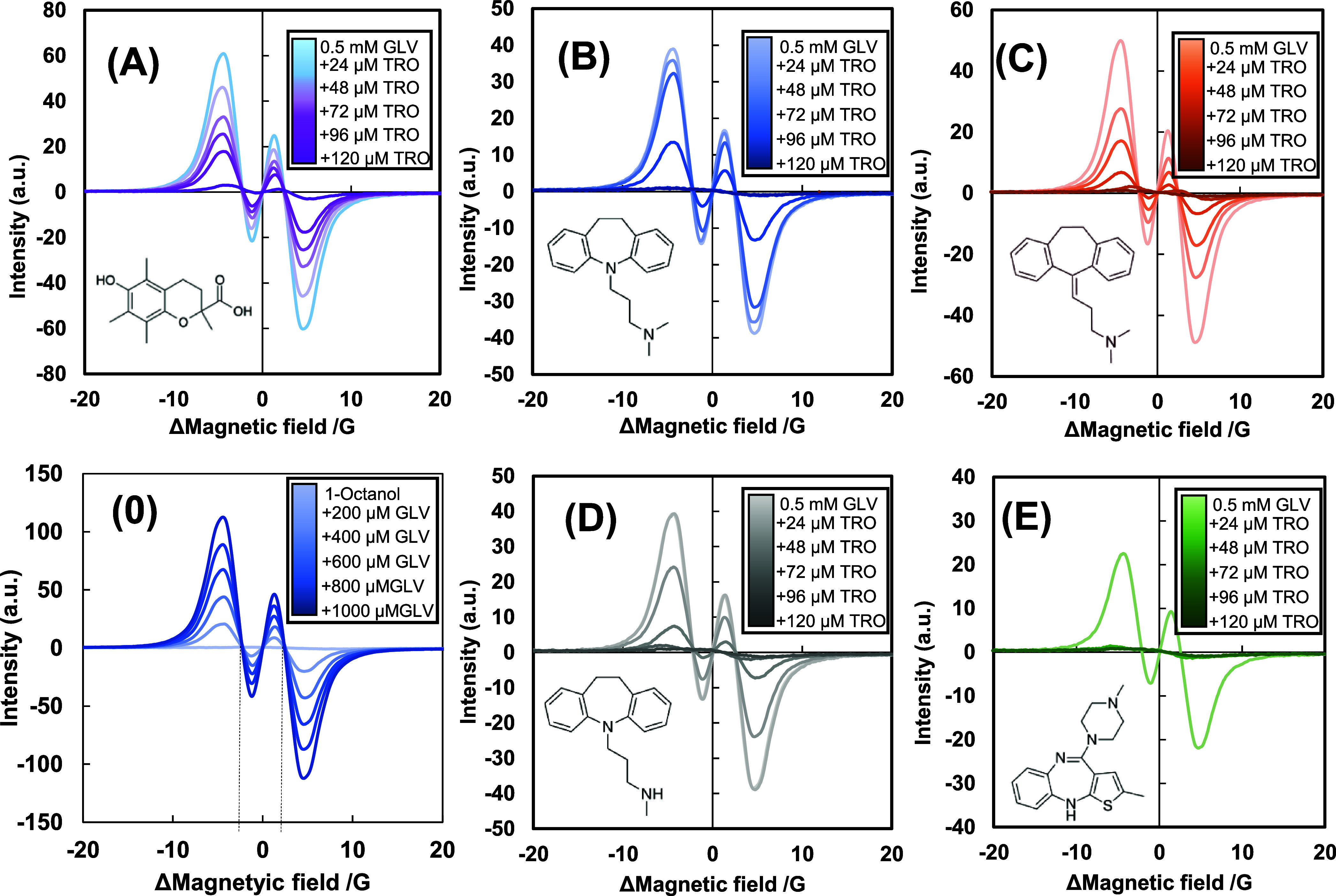
Electron spin resonance spectra of 0.5 mM galvinoxyl (GLV) radicals inhibited by 0–150 μM TRO without (A) or with 2 mM imipramine (B), 5 mM amitriptyline (C), 2.5 mM desipramine (D), and 30 μM olanzapin (E)for 30 min in 1-octanol. The abscissa was aligned to the individual rotatory symmetry point, indicating the magnetic field difference. Hyperfine coupling constants were derived from the abscissa intervals at observed invariable points (peaks of the integrated curve), independently of 0–1.0 mM GLV concentrations on the decrement curve regions in (0), determining as 4.75 G corresponded between the rings-linking methine and aromatic hydrogens.

GLV radicals are hydrophobic reagents containing hydroxyl and oxy-radical moieties shielded by *ortho*-situated *tert*-butyl groups. The EPR spectrum of GLV radicals in an organic solvent is known as a doublet of quintets, of which hyperfine splitting comes from four equivalent hydrogens in aromatic rings and rings-linking methine hydrogen.^77^ However, proper distinction of the parameters will be difficult because of low resolution and signal overlapping.^77^ In the present study, we obtained a stable (for more than 1 h) differential pattern containing two peaks and two troughs for GLV in 1-octanol (Figure 5), with the doublet’s hyperfine coupling constant of 4.75 G. The net reaction progress was measured with a decrease in signal intensity.

Figure 5 presents the EPR spectral decay of 0.5 mM GLV radicals depending on the concentrations of TRO in the absence and presence of 2 mM IMP, 5 mM AMT, 2.5 mM DSP, and 30 μM OLZ. Figure S8 further confirms the GLV radical-scavenging activity of LAs and psychotropic drugs. LDC, but not DBC, decayed GLV radicals at high concentrations, indicate the potential influence of these compounds on GLV behavior. SVD analysis of the entire spectral shape was also employed for the EPR spectra of the GLV radicals (Figures 5 and S8). The SVD parameters summarized in Figure S9 demonstrate that the first and second components are sufficient.

Figure S10 shows that the drug activity, except for that of LDC, was relatively lower than the scavenging of GLV radicals by TRO. The TRO activity (slope of −4.0 × 10^–3^) was insignificantly affected by additions of DBC (−3.3 × 10^–3^), IMP (−3.6 × 10^–3^), AMT (−4.3 × 10^–3^), DSP (−3.9 × 10^–3^), and OLZ (−4.2 × 10^–3^), of which observation differs from the examinations for scavenging DPPH radicals.

Because GLV radicals are water-insoluble, the assay solvent was 1-octanol, which has been frequently used as a biomembrane-mimicking lipid-phase model in Meyers and Overton’s tadpole experiments^34,35^ and Hansch-Fujita QSAR analyses.^70,74^ DBC, IMP, AMT, DSP, and OLZ did not significantly influence the GLV radical scavenging activity of TRO. However, owing to its hydrophilicity, LDC may likely behave as an amphiphilic molecule that forms self-organized micelles or mediates the formation of active oxygen in 1-octanol. Thus, LDC effectively influences the GLV radicals. Despite the hydrophilicity and geometry of OLZ, which differ from those of detergents, the examined concentrations of less than 1/500 exhibited unique characteristics. Therefore, the TRO activity progressed efficiently in the aqueous phase.

TCAs produce reactive oxygen species, a function that photoreactions can potentially induce.^78^ TCAs inducing antioxidant activity produce peroxides, whereas only DSP provides antioxidants.^78^ This distinction in their roles, which might require further confirmation, is a crucial aspect to consider in the present study. The p*I*_50_ values of the drugs and TRO in the absence and presence of the drugs are summarized in Table 1.

### 2.7 Fluorescence Anisotropy Indicating the Restriction of Membranous Fluidity

The psychotropic drugs inhibit lipid peroxidation but reduce the TRO’s inhibitory effect on lipid peroxidation. Meanwhile, these drugs’ radical scavenging activities, except for OLZ, were significantly slight compared to TRO. Therefore, enhancing phospholipid bilayer stabilization may prohibit the invasion of reactive oxygen species, thereby preventing oxidative damage to PUFA moieties. Thus, assuming that TRO scavenging harmful radicals and becoming less noticeable is rational. Subsequently, we examined the effect of the drugs on membrane fluidity to clarify how they protect against oxidative injury.

1,6-Diphenyl-1,3,5-hexatriene (DPH) is a highly hydrophobic fluorescent reagent that enters the hydrophobic phase. When the thermal fluctuations inside the lipids were stabilized, DPH produced intense fluorescence polarization.^79,80^ Fluorescence anisotropy measurements were performed to investigate the effects of psychotropic drugs on the fluidity of the dipalmitoylphosphatidylcholine (DPPC) liposomes.

**Figure 6:**
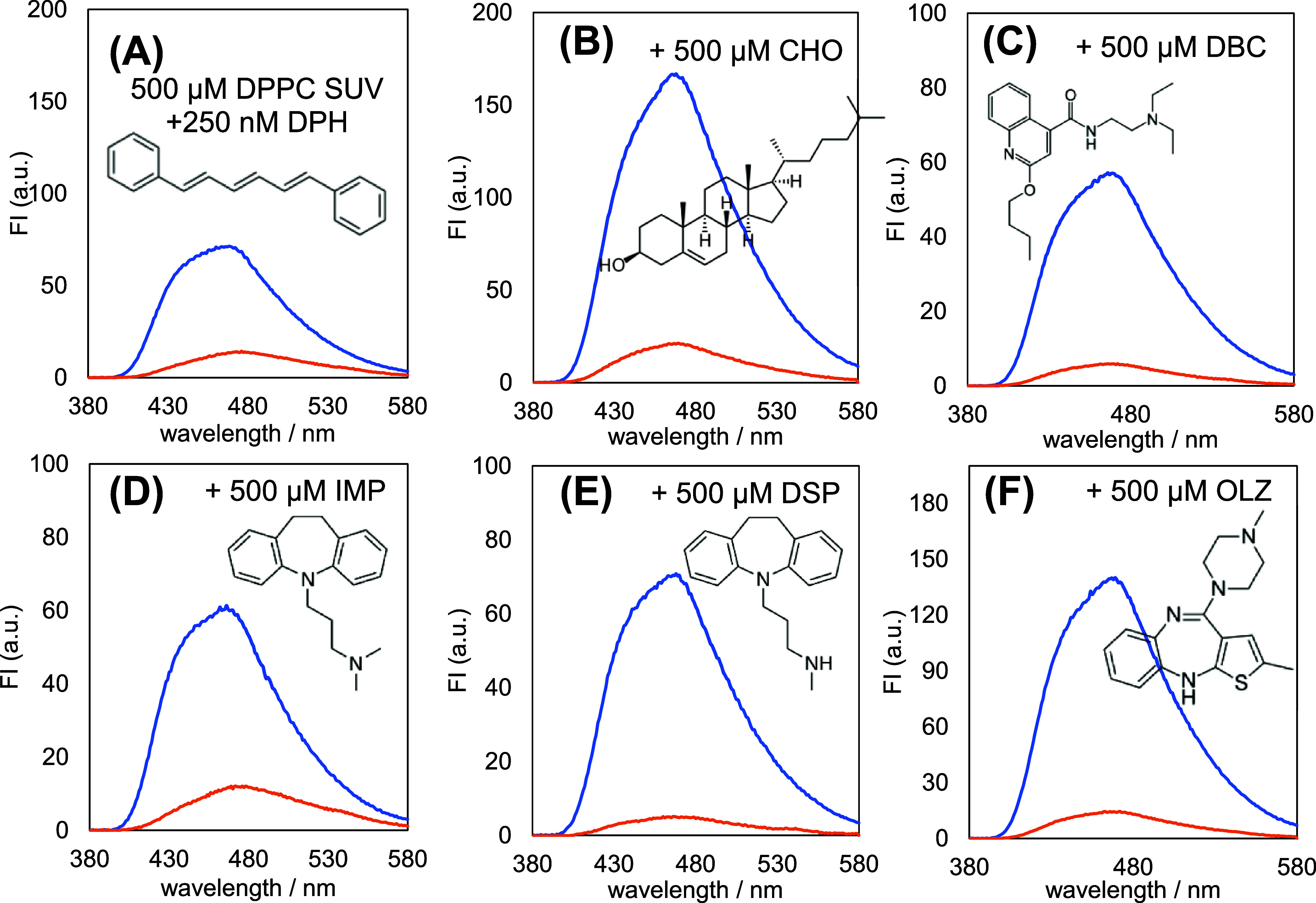
Polarized fluorescence of 1,6-diphenyl-1,3,5-hexatriene doped in the dipalmitoylphosphatidylcholine liposomes with or without 500-μM drugs. Parallel (blue) and perpendicular (amber) polarized fluorescence spectra were measured under the excitation wavelength of 360 nm with slit widths of 20 nm for excitation and fluorescence.

Figure 6 shows the fluorescent spectra of the DPPC liposomes with the phosphate concentration of 500 μM containing 250 nM DPH measured through the parallel (blue) and vertical (amber) polarizers holding over the photomultiplier detector against the vertical polarizer filtering the irradiation lamp. Fluorescence anisotropy is defined as the relative difference between the areas under the curve (AUCs) of the blue and amber spectra, in which the absolute difference is compensated for by the instrumental factor *G* with an observed value of 7.31. Because the DPH molecular fluctuation suppresses fluorescence anisotropy, a slight anisotropy would explain the drastic enhancement of membranous fluidity, including that of DPH. The difference between blue and amber spectra at 500 μM cholesterol (CHO) demonstrates the restraint of the membranous fluidity.

Table 2 summarizes the AUCs calculated from the multicomponent Gaussian integration of the blue and amber spectra and their derived fluorescence anisotropy. The anisotropies determined with and without CHO were 0.32 and 0.024, respectively. The CHO-containing liposomes attenuated the anisotropy by 1/13, confirming that membranous fluidity was the most accelerated.^80,81^ The presence of IMP, DSP, and OLZ decreased the fluorescence anisotropy of DPH. They can improve the membranous flexibility of the lipid bilayers to protect against the invasion of harmful species. The anisotropy with DBC was smaller than that with IMP, DSP, and OLZ. The structural diversity in the two side chains of DBC can facilitate intermolecular interactions, as proposed in our previous study.^57^

**Table 2:** Fluorescence Anisotropy of DPH with and Without the Drugs Organic Compounds Did Not Associate in the Liquid State

	*AUC*_VV_	*AUC*_VH_	*R*
500 μM SUV + 250 nM DPH	6333	357	0.32
+500 μM CHO	14710	1848	0.024
+500 μM LDC	1054	69	0.26
+500 μM DBC	5004	507	0.10
+500 μM IMP	5361	496	0.13
+500 μM AMT	6189	452	0.22
+500 μM DSP	6310	432	0.24
+500 μM OLZ	14180	1403	0.11

As an empirical observation, Walden’s rule stated an invariant fusion entropy Δ_fus_*S*^0^ to be 56.5 J K^–1^ mol^–1^, as long as the organic compounds did not associate in the liquid state. By contrast, we can interpret the remainder of the fusion entropy from Walden’s constant as being assigned to the molecular association between the melting substances in the liquid phase.^58^ Although the absolute value of Walden’s constant is debatable, the fusion entropy difference will reflect a molecular flexibility comparison. Figure S11 shows the drugs’ differential scanning calorimetry (DSC) thermograms. Although the experimentally observed fusion entropy Δ_fus_*S*^0^ was less than Walden’s constant, Table 3 shows the observed values. Structural fluctuations, evaluated using Yalkowski’s log *f*, were also calculated (Table 3). However, the descriptors for the seven-membered ring in DSP and OLZ were insufficient to explain the experimental data. According to the multiple regression analysis, a linear-free energy relationship study was conducted for the fluorescence anisotropy *R* with descriptors of the measured Δ_fus_*S*^0^ and the partition coefficients.
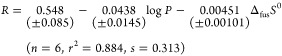


**Table 3:** Physicochemical Properties of the Drugs

	log *P*	Δ_fus_*S*^0^ (J K^–1^ mol^–1^)	log *f*	PSA/MW (Å^2^)	Refractivity (m^3^ mol^–1^)	Polarizability (Å^3^)
LDC	2.26	44.2	2.047	0.1382	73.93	27.77
DBC	4.40	59.1	3.866	0.1586	102.12	40.78
IMP	4.80	46.7	1.592	0.02311	90.61	33.39
AMT	4.92	31.7	1.137	0.01168	101.51	33.34
DSP	4.90	17.6	1.592	0.05732	85.31	31.74
OLZ	4.094	48.5	0	0.09880	93.87	35.35

The QSAR equation indicated that the hydrophobicity and molecular flexibility of the drugs contributed to their effect on membrane fluidity. OLZ, IMP, and DBC inhibited lipid peroxidation and decreased the protective function of TRO membranes by enhancing their fluidity in lipid membranes.

## 3 Discussion

IMP and DSP are dibenzoazepines with butterfly shaped hydrophobic rings and propylamine chains, respectively. The PDB includes three entries of IMP complexes: human serotonin transporter (7lwd), eubacterium leucine transporter (2q72), and Ebola virus glycoprotein (6gqb). The structure of IMP adopted the *equatorial* (*syn*) conformation in 7lwd and 6gqb and showed an *axial (anti)* conformation in 2q72. The DSP complex was published using bacterial leucine transporters (2qb4 and 2qju), and the DSP structure had an *equatorial* conformation. The propylamine chains of IMP and DSP complexed with these transporters were located on the curved ridge side of the hydrophobic rings. Clomipramine (CMP), a 3-chloro derivative of IMP, is retrieved as a complex with the bacterial leucine transporter (2q6h and 2qei) and its complex with the Ebola virus glycoprotein (6g9i). IMP, DSP, and CMP stretched to the interface of the protein pocket and the exterior aqueous phase. Even when we surveyed not only the peptide backbones of proteins but also the individual residues, their propylamine chains were extruded to the exterior. Thus, these dibenzoazepine derivatives tended to adsorb at the interface between the aqueous and hydrophobic phases. DSP and β-lactoglobulin mutant complex (7q19) had a similar nature.

Unlike IMP and DSP, AMT, a dibenzocycloheptenylidene derivative, exhibited unique properties. The ring moiety is connected to the propylamine chain via an exocyclic double bond, which stretches the propylamine chain. AMT complexed with a globular protein (5ha9) and human α1-acid glycoprotein (3apv) have its location inside the hydrophobic pocket of these proteins, with the dimethylamino group which does not expose to the aqueous exterior. The partition coefficient of AMT is comparable to those of IMP and DSP, indicating similar behavior in this aspect (Table 3). However, AMT’s polar surface area (PSA) of AMT is smaller than those of IMP and DSP, which is a unique characteristic that sets it apart. The hydrophobic rings of IMP and DSP contain aniline nitrogen, whereas that of AMT is composed of only hydrocarbons and benzenes, enhancing the hydrophobicity of this moiety. This unique structure allows AMT to easily penetrate the hydrophobic pocket, potentially reaching deep regions of the lipid membrane.

Table 2 compares the interactions of TCAs with the DPPC lipid membrane. Membranous fluidity corresponded to fusion entropies, of which DSP and AMT were less than IMP. The structure of AMT is rigid with an exocyclic double bond; therefore, its mobility in lipids is restricted. This feature might not be suitable for enhancing the membrane flexibility or approaching the polar interface. Although DSP maintains flexibility similar to that of IMP, the DSP fluorescent anisotropy was higher than IMP. Because the PSA of DSP is more significant than those of AMT and IMP (Table 3) and DSP retains amino hydrogen as a hydrogen donor, we considered that its propyl amine chain adsorbs at the polar interface to prohibit mobility. We consider the thioridazine and Ebola virus glycoprotein complex (6g95), in which phenothiazine and 2′-ethylpiperidine moieties were exposed to a polar exterior, and the structure seemed less flexible. By contrast, IMP provides moderate hydrophobicity and molecular fluctuation balance when implementing its fluidity in globular proteins and lipid membranes.

DBC, with its high fusion entropy and broad PSA, exhibits a unique structural profile. Its relatively small partition coefficient suggests localization in the lipid membrane close to the aqueous phase, akin to IMP’s. The structural fluctuation of the flexible alkoxy and alkylamide chains is crucial for facilitating intermolecular interactions.^57,84^ The distinctive properties of DBC and OLZ need to be identified to understand their potential interactions.

Similar to DBC, OLZ exhibits high fusion entropy and a broad PSA with a low partition coefficient. Unlike DBC, OLZ’s structural diversity is not derived from flexible chains, as Yalkowski’s log *f* of zero indicates. Instead, the seven-membered ring with four rotatable bonds allowed for two possible conformations.^83^ The piperadinyl group, with its one *chair* and three *boat* conformations, and the exocyclic bond of nitrogen, which results in *axial* and *equatorial* conformations and two flipping rotamers, contribute to the unique structural diversity of OLZ. This resulted in a minimum of 2 × 4 × 2 × 2 = 32 identifiable conformations, indicating that its flexibility was not poor, which aligns with the high fusion entropy. The molecular structural reason for OLZ’s slight fluorescence anisotropy would be shared with that of DBC, further highlighting its unique properties.

Our study revealed an interesting contrast between the structural flexibilities of OLZ DBC and AMT. Although complexes of OLZ, QTP, and DBC with proteins could not be determined, the PDB includes entries of the CLZ/histamine H4–receptor complex (8jxv) and deschloro-clozapine/muscarinic M4–receptor complex (8e9x and 8e9w). These olanzapine analogs intrude into the hydrophobic pockets of proteins. OLZ and DBC approach the protein’s hydrophobic pocket and deep regions in lipid membranes. The notable structural flexibility observed in OLZ and DCB is significant compared with that in AMT.

In Table 3, refractivity and polarizability represent the density and dipole moment, respectively; the former is proportionally correlated with the partition coefficient.^70,74^ Polarizability correlates with anisotropy with determination coefficient *r*^2^ = 0.694, although the multiple regression analysis appending other descriptors did not improve. These static parameters did not statistically characterize the fluorescence anisotropy of the drugs in the present study.

Table 1 demonstrates that the drugs slightly influenced TRO’s p*I*_50_ against DPPH and GLV radicals. For the drugs’ p*I*_50_ against DPPH and GLV radicals, OLZ yielded the highest value (4.2 and 4.1) comparable to TRO (4.0 and 4.1). The effects of IMP, AMT, DSP, and OLZ on lipid peroxidation in liposomal membranes resembled the radical-scavenging activities of DPPH and GLV. However, because OLZ and DBC were the highest and lowest outliers, respectively, the regression analysis may have poor statistical significance.

The slope dp*I*_50_^Drug^/d[TRO] in Figure 3A indicates how TRO inhibited the inhibitory effect of the drug on lipid peroxidation. The slope of the hydrophilic LDC was small, whereas those of AMT and DSP were large. The slope values were correlated to the fusion entropy Δ_fus_*S*^0^ with the determination coefficient *r*^2^ = 0.829 but not to the partition coefficient log *P* (*r*^2^ = 0.311) and fluorescence anisotropy (*r*^2^ = 0.172). Further, multiple regression analyses yielded the following QSAR equation:
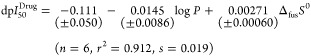

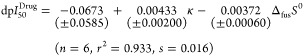


We consider that fusion entropy reflects molecular fluctuations. Here, κ is polarizability. Polarizability was more favorable than hydrophobicity for representing the drug’s accelerated TRO activity. This descriptor suggests that TRO in liposomal membranes influences the drug’s molecular electronic localization/reactivity or lipid membranous depth relative to the peroxidation site of polyunsaturated hydrocarbons in phospholipids.

Our study of the TRO’s p*I*_50_ for lipid peroxidation, as indicated by the slope dp*I*_50_^TRO^/d[Drug] in Figure 3B,C, is novel and intriguing. This revealed the inhibitory effects of TRO on lipid peroxidation. The LDC, DBC, and AMT results were slightly unstable concentration-dependent. However, IMP and DSP were amplified by approximately 10–20 fold to LAs, whereas OLZ enhanced potency by 50–80 fold. Thus, the drugs did not influence the radical scavenging activity of TRO but enhanced membrane fluidity and stability. Moreover, TRO protects the lipid membrane from injury due to reactive oxygen species generated by Fenton’s reaction. If the drugs stabilize the lipid membranes, the lipid phase excludes both the reactive oxygen species and TRO. The drugs then inhibit lipid peroxidation and simultaneously impede TRO activity. Thus, our findings suggest the phenomenon of lipid membranes induced by TRO and drugs.

LDC cannot affect TRO’s activity because its membranous fluctuation was insufficient. Although DBC can modify membrane fluidity, its hydrophobicity is insufficient for contact with lipid peroxidation sites in the deep region of the membrane. IMP behaves flexibly and reaches the depth of the hydrophobic lipid peroxidation sites. AMP can also approach these sites, although it has poor flexibility. DSP might induce effective perturbation but is adsorbed on the aqueous interface. OLZ was comparable to IMP but possessed potent radical scavenging activity; therefore, OLZ and TRO could compete or concord to reduce reactive oxygen species. Thus, antidepressants and MARTA antipsychotics inhibited liposomal lipid peroxidation because of membranous stabilization caused by enhanced lipid bilayer fluidity. This clarifies how TCAs inhibit oxidative stress.

Although the inhibitory activities of SRT, FLX, PRX, DHM, and diazepam (inactive, data not shown) on lipid peroxidation were screened (Figure 2 and Table 1), these results were not as potent as those of IMP. However, this does not deny the potential therapeutic applications of these drugs. We canceled the competition assays using TRO. Nevertheless, the fact that SRT, FLX, and PRX, which are selective serotonin reuptake inhibitors (SSRIs) in complexes with the human serotonin transporter (7txt, 6awp, and 6dzw), were stored in the hydrophobic binding site is intriguing. SSRIs have high individual specificity; however, they do not share the structural features of drugs. Therefore, they are unsuitable for the comparative approach used in this study.

TCAs such as IMP, DSP, and AMT inhibit the presynaptic reuptake of noradrenaline and serotonin and stimulate histaminergic H1, muscarinic, and presynaptic α1-adrenergic receptors. The monoamine hypothesis of depression, which formulated that depression was associated with a deficiency in the transmission of serotonin, noradrenaline, and dopamine, led to the development of SSRIs and serotonin–noradrenaline reuptake inhibitors. In addition to restoring regular function, antidepressants are effective in other psychotic disorders, such as panic disorder, obsessive-compulsive disorder, and bulimia. However, the enhancement of serotonergic or noradrenergic transmission is not consistently effective in depression. Conventional TCAs are sometimes more effective than improved transporter-selective inhibitors. As the pharmacological activity does not necessarily match the potencies of the competitive affinity experiments and protein complex formations, the essential keys of the activity may not yet have been identified for the serotonin transporter for IMP and the muscarinic receptor for CLZ. The present study proposes that the sites of LAs, IMP, and OLZ action possess the possibility of being lipid membranes.

As a problem with the monoamine hypothesis, the latency of the response to antidepressants is refractory. The receptor model may explain pathogenesis, although this remains debatable. As an alternative proposal, the neuroplasticity hypothesis describes how the morphological changes in hippocampal neurons, such as the shortening length of dendrites and the decreased number and density of spines, reduced hippocampal volume. We considered that not only the shapes of neurons but also the physicochemical properties of neuromembrane lipids, namely, membranous fluidity, would be contributing. Serotonin and noradrenaline are modulated by the levels and activities of reactive oxygen and nitrogen species, and oxidative stress processes may be relevant in the pathogenic mechanisms underlying many major psychiatric disorders.^71,73,84^ This would be therapeutically advantageous, as TCAs protect the membrane from reactive oxygen species attack, and OLZ can inhibit lipid peroxidation.

Marine toxins induce the latency of neurological symptoms after being undertaken and are spread by bioconcentration; ciguatoxins cause the most victims a year worldwide.^82−84^ Neurological symptoms are the most distinctive and enduring feature of ciguatoxin poisoning, with effects lasting <1–48 h and weeks to months.^85−87^ Unlike the monoamine hypothesis, ciguatoxin behavior cannot be considered to be caused only by its concentration around neurons. Marine toxins would accumulate in biomembranes beyond the life cycle of receptor proteins and are predated by individuals. This could have unexpectedly induced neurological symptoms. The receptor model paradigm assumes that the discovery of the site of action leads to the resolution of any therapeutic issue; however, this is not consistent with the goal. Furthermore, as identifying the site of action, that is, the drug-receptor, provides genetic approaches, we could lose its cooperation convenience without the receptor model. The binding of stimulants/inhibitors to a specific site induces the related physiological system. However, this is not an antonym for a disease or a disorder.

CMP shows promise in preventing brain damage caused by direct SARS-CoV-2 infection and the indirect impact of viral infection through an excessive inflammatory response.^88,89^ Although the underlying mechanisms require further studies, the significant inhibition of the Ebola virus, SARS-CoV, and MERS-CoV replication demonstrated by CMP instills confidence.^88,89^ This study might include the potential therapeutic uses of TCAs and OLZ in preventing the detrimental effects of viral infections.

## 4 Conclusion

LDC and DBC effectively inhibited lipid peroxidation in the EyPC liposomal membrane induced by Fenton’s reaction despite their lack of DPPH and GLV radical scavenging activity. As a novel finding, we also discovered that conventional antidepressants such as IMP, DSP, and AMT share similar features. Furthermore, OLZ, a MARTA-type antipsychotic drug, exhibits radical-scavenging activity and inhibits lipid peroxidation at significantly lower concentrations than LAs and TCAs. However, both TCAs and OLZ reduced the protective function of TRO in the liposomal membranes. Fluorescence anisotropy results demonstrated that DBC, IMP, and OLZ enhanced the fluidity of the DPPC membranes. Thus, antidepressants and MARTA antipsychotics inhibited liposomal lipid peroxidation and reduced the TRO protective function of membranes, owing to membranous stabilization caused by the enhancement of lipid bilayer fluidity.

Despite the different presynaptic uptake and postsynaptic receptor targets of TCA and MARTA auxiliary drugs, they stimulate histaminergic H1, muscarinic, and presynaptic α1-adrenergic receptors. Conventional TCAs are occasionally more effective than improved transporter-selective inhibitors such as SSRIs. We hypothesized that the neuroplasticity, physicochemical properties of the neuromembrane, and membrane fluidity would be contributory factors. This would be therapeutically advantageous, as TCAs protect the membrane from reactive oxygen species attack, and OLZ can inhibit lipid peroxidation. The receptor model assumes that the discovery of the site of action leads to the resolution of any therapeutic issue; however, this is not consistent with the goal. Furthermore, by identifying the site of action, the drug receptor, which provides genetic approaches, we could lose its cooperation convenience without the receptor model. The binding of stimulants/inhibitors to a specific site induces a physiological response. However, this is not an antonym for a disease or a disorder. These may be possible in general, and LAs and neurotropics may act on nervous membranes. Therefore, the effects of lipid membrane-acting drugs on biomembranes require reevaluation.

## 5 Materials and Methods

### 5.1 Materials

BHT (CAS RN 128–37–0), CHO (57–88–5), DBC (85–79–0), DPPH (1898–66–4), DHM (58–73–1), DPPC (63–89–8), FLX (54739–18–3), GLV (2370–18–5), LDC (137–58–6), OLZ (132539–06–1), PRX (61869–08–7), SRT (79617–96–2), TBA (504–17–6), TRO (53188–07–1), and EyPCs were purchased from Tokyo Chemical Industry (Tokyo, Japan). AMT (50–48–6), DSP (50–47–5), IMP (50–49–7), and 1,1,3,3-tetraethoxypropane (122–31–6) were purchased from FUJI-FILM Wako Pure Chemical Corporation (Osaka, Japan). All other reagents used were of the highest commercially available grade. Phosphate-buffered saline (PBS) was prepared as an aqueous solution containing 140 mM NaCl, 8.9 mM Na_2_HPO_4_, and 1.5 mM KH_2_PO_4_, adjusted to pH 7.4, using 1 M NaOH-*aq* and 1 M HCl-*aq*.

### 5.2 Preparations of EyPC, DPPC, and CHO-Containing DPPC Liposomes

According to Bangham’s method, phospholipids were dissolved in diethyl ether and evaporated for 90 min to form a lipid film inside the flask. The films were dried in a desiccator for 18 h. The dried lipid film was swollen in PBS by spontaneous hydration and vigorously stirred on a shaker at 300 rpm at 323 K for 30 min. Due to self-organization, the lipid film fragments were dispersed as multilamellar vesicles (MLVs). The MLV suspension was fractured with a sonication probe at 50% amplitude with a 50% duty cycle for 60 min, sustaining as liposomes of SUVs.^51^ Total and organic phosphorus concentrations were quantified using phosphor–molybdate complex chronometry at a wavelength of 800 nm.^51^ The DPPC liposomes containing no unsaturated acyl chains and the CHO-spreading DPPC liposomes were prepared and evaluated similarly, following the same steps.

### 5.3 DLS in Measuring Particle Diameters and Distributions

DLS analyzed the MLVs and SUVs at 300 K with an ELSZ-2000ZS instrument (Otsuka Electronics Co., Ltd., Osaka, Japan). Vesicles dispersed in solution are subjected to Brownian motion, the rate of which depends on the particle diameter. Illuminating the moving vesicles with laser light produces scattered light fluctuations, which enable the calculation of hydrodynamic diameters and distributions.

### 5.4 Lipid Peroxidation

Lipid peroxidation in liposomes (phosphorus concentration 1.3 μg/μL) was induced by adding Fenton’s reagent containing 0.2 mM Fe(NH_4_)_2_(SO_4_)_2_ and 0.1 mM H_2_O_2_ at 310 K in PBS–ethanol mixture at a ratio of 9:1 for 12 min.^51^ Hydroxide radicals injure EyPC liposomal lipid membranes and produce peroxide products. After the appropriate period, adding 2% w/v BHT terminated lipid peroxidation. Generally, the obtained peroxide products were evaluated using the TBARS chromometric assay at a peak wavenumber of 530 nm.^51^ Because the TBA chromogenic reaction of 1,1,3,3-tetraethoxypropane proportionally produces TBARS from MDA derivatives, the calibration curve is presented as a diagram of the absorbance of the converted amount of MDA. In the present study, TBARS spectra in 400–700 nm were measured using a V-750 UV–vis spectrophotometer (JASCO, Japan) at room temperature. TBARSs from MDA and lipid peroxidation products showed spectra with a single peak at 530 nm and twin peaks at 455 and 530 nm, respectively.^51^ However, the intensity of the 455 nm peak irregularly changed with additives, thus computing SVD for noise elimination in the 530 nm peak.

### 5.5 Electron Paramagnetic Resonance (EPR) Measurement

EPR was measured according to the method described by Takatsuka et al.^60^ EPR spectra were recorded in the X band (9.72 GHz) using a micro EPR spectrometer (Bruker, Germany) 30 min after mixing the sample with each radical species in a quartz capillary at 298 K. The media were 95% ethanol, water mixture at a volume ratio 4:1 for DPPH assay, and 1-octanol for GLV assay. To examine the inhibitory effects of drugs on the TRO’s radical scavenging (Figures 4 and 5), 100 μL of TRO and 200 μL of drug stock solutions were mixed, and 200 μL of radical species stock solution was added. The reaction solution finally contained 0.5 mM DPPH or GLV radicals with appropriate concentrations (0–150 μM) of TRO with or without 16 mM LID, 10 mM DIB, 2 mM IMP, 5 mM AMT, 2.5 mM DSP, or 30 μM OLZ. To determine the radical-scavenging activity of the drug (Figures S7 and S10), radical species were added to the drug solutions at the appropriate concentrations. Without drugs, the hyperfine coupling constants were derived from the intervals of the observed invariable points (corresponding to the peaks of the integrated curve) independent of the TRO concentration in the decrement curve regions (Figures 4 and 5).

### 5.6 DSC and Estimating the Entropy of Fusion

The melting temperatures *T*_m_ and fusion enthalpy changes Δ_fus_*H*^0^ were measured by DSC (DSC8230, Rigaku Co., Tokyo, Japan). The sample of 10 mg was placed in a closed aluminum pan and performed at a temperature scanning speed of 10 K min^–1^ under a nitrogen gas flow rate of 30 cm^3^ min^–1^.^80^ The *T*_m_ was defined as the intersection between the tangent consistent with the gradient at the inflection point of the exothermic curve left side and baseline, and the Δ_fus_*H*^0^ was obtained as the numerical integration of the DSC thermogram.^70,71^ According to Klausius’ definition, the fusion enthalpy changes Δ_fus_*H*^0^ were calculated as the Δ_fus_*H*^0^ divided by the *T*_m_.^64−69^

### 5.7 Fluorescence Anisotropy Measurement

The DPPC lipid film was doped with DPH at a molar ratio of 200:1, preparing the SUV liposomes, in which the final concentration of DPPC was measured at 500 μM in the measured suspension. Each drug at the final concentration of 500 μM was added to the DPH-doped DPPC liposomal suspension. Fluorescence anisotropy was measured using a Shimadzu RF-5300PC spectrofluorophotometer (Shimadzu, Kyoto, Japan) at standard temperatures under excitation and fluorescence excitation wavelengths of 360 and 20 nm, respectively.^78^ The solvent was a mixture of PBS and 10% EtOH. Steady-state fluorescence anisotropy *R* was defined as
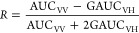
1where *AUC*_VV_ is the total area under the DPH fluorescence spectral curve when both the excitation and emission polarizers are vertical. Meanwhile, *AUC*_VH_ is the corresponding value when the excitation and emission polarizers are perpendicular to vertical and horizontal, respectively. The instrumental coefficient *G* is the ratio of the photomultiplier’s sensitivity for vertically and horizontally polarized light, which is derived from the observed detections for vertically and horizontally polarized light, given by the practical vertical to horizontal components, because the excitation light is polarized owing to the diffraction grating, where *G* = *AUC*_HV_/*AUC*_HH_ = 7.31.^79^

### 5.8 SVD Procedure

The SVD is based on linear algebra.^58−69^ The SVD was used to analyze the observed spectra using the previously described techniques. The *i*-th spectrum {Φ⃗_*i*_|1≤ *i* ≤ *n*} is represented as an *m*-dimensional vertical vector composed of signals represented by functions of the sampling wavelength {*w*_*j*_|1≤ *j* ≤ *m*}. Matrix M comprises a horizontal sequence of vectors from the first spectral vector through the *i*-th spectral vectors, which contain the calibration spectral vectors, with an *m* by *n* rectangular matrix that is defined as follows, where *m* ≥ *n*:
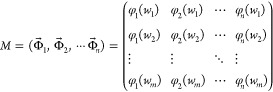
2where M and M*^t^* denote the real and transposed matrices, respectively. Their products, M*^t^*M and MM*^t^* become orthogonal matrices, and the eigenvectors are the rows of Ψ and Λ, respectively. The matrices describing M are transformed into the following formulas:
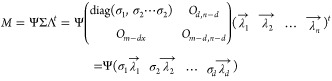
3

In descending order, the diagonal matrix Σ represents the diagonal elements {σ_i_ |1≤ *i* ≤ *d*} of the positive absolute values. These elements are singular values, indicating dispersion.^58−67^ The *i*-th column of the orthogonal matrix Λ is the coefficient vector corresponding to the singular value *σ*_*i*_ and vector *λ⃗*_*i*_ is known as a singular vector. Matrix Ψ consists of rows that are the basis function vectors.^76^ We practically determined the dimensionality according to the diagram for the logarithm of the singular values in descending order versus the spectra, that is, the minimum dimensionality of the basic functions required to reproduce the vector space of the spectra. This could be unimportant with a singular value of less than several hundred times the highest singular value of the first principal component. Because the dimensionality *d* is determined under this criterion instead of the mathematical rank ρ, the yielded principal components approximately reproduced the vector space, including the spectrum as the *j*-th feature vector *x⃗*_*j*_ composed of the *i*-th elements *x*_*i,j*_:
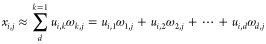
4

Finally, the sum of the first and second components, ω, was defined and used in the following equation.
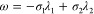
5

The obtained ω values of the calibration spectra for standard MDA-TBARS, DPPH, or GLV were converted as the molarity.^51^ For ESR spectra, the −σ_1_λ_1_ vectors were sufficient to represent the observed spectra.^60^

### 5.9 Modeling of Protein–Drug Complexes

The three-dimensional structures of the proteins and their drug-binding complexes were surveyed by X-ray diffraction crystallography from the RCSB PDB via the PDBj Web site (Institute for Protein Research, Osaka University). The ligand-binding proteins were projected as CPK or ball-and-stick models onto the backbone tube models using the WebGL-based molecular viewer Molmil. The crystalline coordinates of small organic compounds were obtained from the Cambridge Crystallographic Data Center.

## Supporting Information Available

**Figure S1**. TBARS spectral references of MDA derived from various concentrations of the standard 1,1,3,3-tetraethoxypropane, EyPC liposomes at the concentration of 1.3 μg/μL represented as organic phosphate amounts, and TRO solutions without the SUVs. **Scheme S1**. SVD procedure divides M into three matrices: ψ, Σ, and transposed Λ. **Figure S2**. Singular values (σ*_i_*) and basis functions (ψ*_i_*) were obtained due to the SVD processing for the matrix containing the obtained spectra, including the calibration spectra. **Figure S3**. The first, second, and third singular vectors for IMP, AMT, DSP, and OLZ are computed by the SVD processing for the obtained spectra. Diagrams between the negative logarithm of the SVD component score change (ω–ω_∞_) relative to the maximum range (ω_0_-ω_∞_) and the concentrations of 0–3.2 mM LDC, 0–2 mM DBC, 0–0.5 mM TCAs and DHM. **Figure S4.** Diagrams between the negative logarithm of the SVD component score change (ω–ω_∞_) relative to the maximum range (ω_0_-ω_∞_) and the concentrations of 0–0.4/0.5 mM TCAs and DHM. **Figure S4**. Diagrams between the negative logarithm of the SVD component score change (ω–ω_∞_) relative to the maximum range (ω_0_-ω_∞_) and the concentrations of OLZ, IMP, DSP, and AMT. Additionally, those to the TRO concentrations under OLZ, IMP, DSP, and AMT. **Figure S5**. The ESR spectra of DPPH radicals inhibited by LDC, DBC, IMP, AMT, DSP, and OLZ for 30 min in a 71% ethanol and water mixture. The abscissa was aligned to the individual rotatory symmetry point, indicating the magnetic field difference. **Figure S6**. Singular values (σ*_i_*) and basis functions (ψ*_i_*) were obtained as a result of the SVD processing for the matrix containing the obtained spectra, including the calibration spectra. The first component ψ_1_ and the second component ψ_2_ include ±2.5, ± 10, and ±20 G signals, whereas the third component ψ_3_ seems to be irregular noise. As the singular vectors λ_1_ and λ_2_ are correlated with each other, we considered the first component as the products of ψ_1_, σ_1_, and λ_1_ would reproduce the observed spectra.^60^**Figure S7**. Diagram of DPPH radical consumption to the TRO concentrations in the presence of drugs and those to the TCAs concentrations and OLZ concentrations without TRO. **Figure S8**. The ESR spectra of GLV radicals inhibited by LDC, DBC, IMP, AMT, DSP, and OLZ for 30 min in 1-octanol. The abscissa was aligned to the individual rotatory symmetry point, indicating the magnetic field difference. **Figure S9**. Singular values (σ*_i_*) and basis functions (ψ*_i_*) were obtained as a result of the SVD processing for the matrix containing the obtained spectra, including the calibration spectra. The first component ψ_1_ includes ±4.6 G signals, whereas peaks and troughs of the second component ψ_2_ and the third component ψ_3_ seem to differ from the obtained spectra. As the singular vectors λ_1_ and inversed λ_2_ are correlated with each other, we considered the first component as the products of ψ_1_, σ_1_, and λ_1_ would reproduce the observed spectra.^60^**Figure S10**. Diagram of GLV radical consumption to the TRO concentrations in the presence of drugs and those to the TCAs’ concentrations, OLZ’s, and LDC’s without TRO. **Figure S11**. DSC thermograms for IMP, AMT, DSP, and OLZ observed melting temperatures of 173.5, 196.9, 217.5, and 194.7°C, respectively (PDF)

## Abbreviations

5HT: 5-hydroxytryptamine (serotonin)
AAP: atypical antipsychotics
ABTS: 2,2′-azinobis(3-ethyl-2,3-dihydrobenzo-thiazole-6-sulfonic acid) radical cation
AMT: amitriptyline
BDZ: 1,4-benzodiazepine derivative
BHT: 2,6-di-*tert*-butyl-*p*-cresol
CLZ: clozapine
CMP: clomipramine
CYP: cytochrome P450
DBC: dibucaine
DHM: diphenhydramine
DLS: dynamic light scattering
DPH: 1,6-diphenyl-1,3,5-hexatriene
DPPH: 2,2-diphenyl-1-picrylhydrazyl radical
DSP: desipramine
DSS: dopamine system stabilizer
EyPC: hen egg yolk lecithin
FLV: fluvoxamine
FLX: fluoxetine
GABA: γ-aminobutyric acid
GLV: galvinoxyl radicals
IMP: imipramine
LA: local anesthetic
LDC: lidocaine
LFER: linear free energy relationship
MALTA: multiacting receptor-target antipsychotics
MDA: malondialdehyde
MLV: multilamellar vesicle
OLZ: olanzapine
PBS: phosphate buffered saline
PDB: the Protein Databank
PRX: paroxetine
PSA: polar surface area
PUFA: polyunsaturated fatty acid
QSAR: quantitative structure–activity relationships
QTP: quetiapine
RCSB: the Research Collaboratory for Structural Bioinformatics
SDA: serotonin-dopamine antagonists
SDAM: serotonin-dopamine activity modulator
SDS: sodium lauryl sulfate
SRT: sertraline
SSRI: serotonin selective receptor inhibitor
SUV: small unilamellar vesicle
SVD: singular value decomposition
TAP: typical antipsychotics
TBA: thiobarbituric acid
TBARS: TBA reacting substance
TCA: tricyclic antidepressants
TRO: 6-hydroxy-2,5,7,8-tetramethylchroman-2-carboxylic acid (trolox)
Cho: Cholesterol
