# Supplementary material for: Reactivity of Olanzapine and Tricyclic Antidepressants on the Protective Effects of Trolox on Lipid Peroxidation Evaluated Using Fluorescence Anisotropy, Electron Paramagnetic Resonance Spectrometry, and Thermal Analysis
Source: ACS Chem Neurosci. 2025 Jan 17;16(3):462–78. doi: 10.1021/acschemneuro.4c00702 (PMC11809279; doi:10.1021/acschemneuro.4c00702)

Reactivity of Olanzapine and Tricyclic Antidepressants on the Protective Effects of Trolox on Lipid Peroxidation Evaluated using Fluorescence Anisotropy, Electron Paramagnetic Resonance Spectrometry, and Thermal Analysis

[Supporting Information]

Authors:
Yusuke Horizumi[1]; Reo Tanada[1]; Yuya Kurosawa[1]; Miwa Takatsuka[1]; Tomohiro Tsuchida[1]; and Satoru Goto*[1]

Affiliation:
[1]Faculty of Pharmaceutical Sciences, Tokyo University of Science, 2641 Yamazaki, Noda, Chiba 278-8510, Japan

*To whom correspondence should be addressed at s.510@rs.tus.ac.jp

CONTENTS

TBARS Spectra of EyPC Lipid Peroxidation ………………………………… S2
Quantitative Evaluation of the Peroxide Peak Intensity Using the Singular Value Decomposition (SVD) Procedure …………………………………………….. S3
Quantitative Evaluation of the DPPH Radical Scavenging Activity of TRO in the Presence of Drugs Using the SVD Procedure …..…………………………….. S10
Quantitative Evaluation of the GLV Radical Scavenging Activity of TRO in the Presence of Drugs Using the SVD Procedure…………………………………………… S13
Thermal Analyses for IMP, AMT, DSP, and OLZ ……………………………... S16
Appendix-1 (exposing at the protein/water interface) …………………………. S17
Appendix-2 (exposing at the protein/water interface) …………………………. S18
Appendix-3 (exposing at the protein/water interface) …………………………. S19
Appendix-4 (exposing at the protein/water interface) …………………………. S20
Appendix-5 (exposing at the protein/water interface) …………………………. S21
Appendix-6 (exposing at the protein/water interface) …………………………. S22
Appendix-7 (exposing at the protein/water interface) ……………..…………... S23
Appendix-8 (intruding into the membrane domain) ……………..…………….. S24

Thiobarbituric Acid Reacting Substance (TBARS) Spectra of Egg-york Phosphatidylcholine (EyPC) Lipid Peroxidation

The sample solutions prepared in phosphate-buffered saline (PBS)-ethanol mixture at a ratio of 9:1 were induced peroxidation by the addition of Fenton's reagents (FR), containing 0.2 mM $Fe(NH_4)_2(SO_4)_2$ and 0.1 mM H_2O_2 at 310 K. After 12 min, adding 2% w/v BHT terminated lipid peroxidation. The TBA chromogenic reactions were carried out as described in Section 5.4.

Figure S1 shows the TBARS spectra in reference experiments. In Figure S1A, the TBARS spectra for 0-2.0 μM malondialdehyde (MDA) as a standard substance expressed a single peak at 530 nm and shoulder at about 500 nm. According to the peak separation analysis, shoulders at 500 nm were proportional to the 530 nm signals in Figure S1A. In Figure S1B, the small unilamellar vesicles (SUVs) of EyPC liposomes (at the incinerated phosphate concentrations of 0-15 μM) peroxided using the FR peroxide showed the TBARS spectra with the twin peaks at 455 nm and 530 nm, in which the former gradually increased but the latter conspicuously elevated. In Figure S1C, the mixture of 0-15 μM trolox (TRO) and the FR also gave the twin peaks, in which the 455-nm peak's intensity decreased and the 530-nm peak's intensity enhanced, depending on the TRO concentration. The independence of the reference oxidative product MDA on the 455-nm peak proved that the oxidative by-product of liposomal lipids and TRO induced this peak. These results indicated that the co-occurrence between the 455-nm and 530-nm peaks changed, corresponding to the substances to be reacted in the TBA chromogenic reaction.

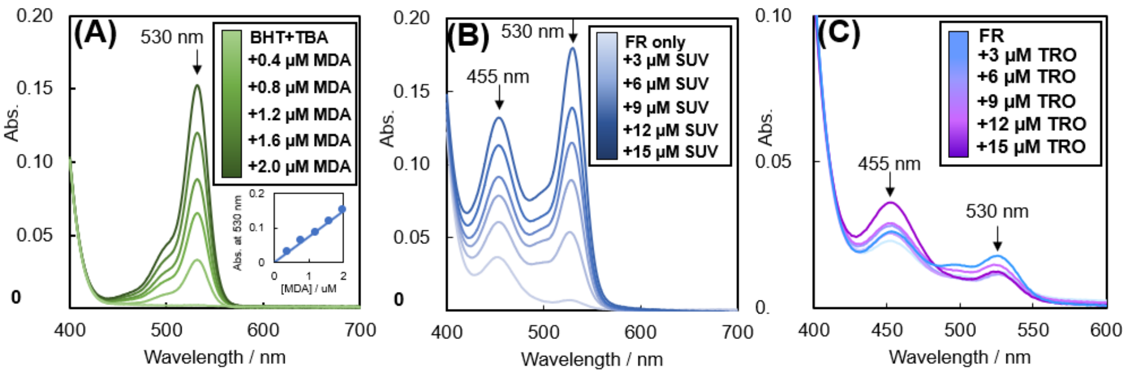

Figure S1. TBARS spectral references of (A) MDA derived from various concentrations of the standard 1,1,3,3-tetraethoxypropane, (B) EyPC liposomes at the concentration of 1.3 μg/μL represented as organic phosphate amounts, and (C) TRO solutions without the SUVs.

Quantitative Evaluation of the Peroxide Peak Intensity Using the Singular Value Decomposition (SVD) Procedure

Lipid peroxidation provided the MDA (OHC-CH_2-CHO) and other peroxidized products in fixed ratios, and the non-MDA peroxides induced the TBARS with its spectral peak at 455 nm. Figure S1B indicates a certain proportion in the lipid constitution of the present liposomes. In contrast, TRO was used as a reagent in this experiment, and its TBA chromogenic reaction produced the ingredients with absorption at 455 nm and 530 nm. This means that the 530 nm absorbance contains the signals of MDA, the related peroxide, and reagents. To extract the MDA-relating signal from the TBARS spectra, we attempted the singular value decomposition (SVD) procedure, generally used to refine the significant signals from random or instrumental noise.

The matrix M for the obtained spectral data is a horizontally sequential row of the column vectors (m=301) for the obtained spectral data demonstrated in Figures 1 and S1 (n=6×6×4+6×3=162). As shown in Scheme S1, the SVD procedure processes the M to derive the product of three matrices: i.e., the rectangular matrix Ψ containing the basis vectors (basis functions) ψi, the diagonal matrix Σ consisting of the singular values σ_i in descending order, and the transposed rectangular matrix Λ^t for the singular vectors λ_j. The first basis function (as a column) reveals the averaged spectrum for all samples, and the continuous basis functions represent the differential spectra depending on the experimental conditions for measurements. The observed spectra are reproduced or approximated by the linear combinations of the basis functions multiplied by the corresponding singular values and vectors.

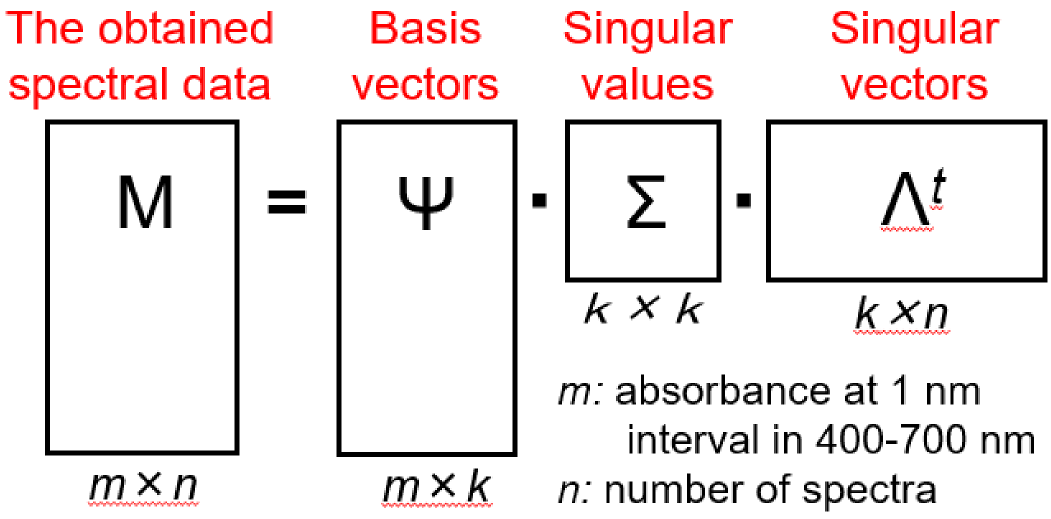

Scheme S1. SVD procedure divides M into three matrices: ψ, Σ, and transposed Λ.

Figure S2A shows the SVD computation provided the basis functions ψi, singular values σ_i, and singular vectors λ_i. Rank is a label of the observed sample, being less or equal to n=162. Singular values σ_i are in descending order, and the most significant value corresponds to the average spectrum of the first basis vector. Statistically, the cumulative amount of singular values σ_i represents the variance in reproduction for the approximated spectrum. The cumulative ratio up to the third singular value (σ_3=1.37; 8% to σ_1=16.6) was 90.9%, leading to the rank of 3 sufficient for reproduction.

Figure S2B shows the spectra extracted as the basis functions are demonstrated. The first basis function (blue) is flip-flapped, representing the average spectrum containing 455-nm and 530-nm peaks (troughs). The second basis function (amber) has a negative peak at 455 nm and a positive peak at 530 nm (with about 500 nm shoulder), which seems to reflect the antiparallel contributions in the 455-nm peak for non-MDA by-products in lipid peroxidation and additives (TRO and others), as shown in Figure S1. The third basis function (gray) has negative peaks at 455 and 530 nm and foothills under the wavelength of 425 nm. As it would not accord to the continuous change in the lipid amount-dependent spectral change (Figure S1B), we considered that indicating the difference among the experimental spectral series. The observed spectra would be reproduced with linear combinations of ψ_1 and ψ_2. Adding an adequately magnified ψ_2 curve to the flipped ψ_1 curve could increase the 530-nm signal and decrease the 455-nm signal, reproducing any observed spectra.

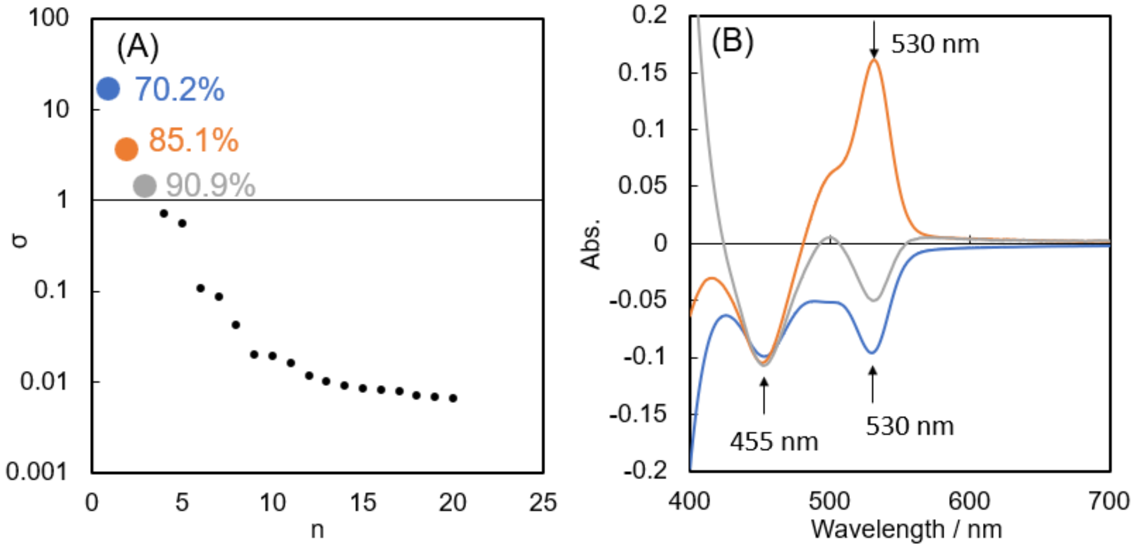

<u>**Figure S2**. (A) Singular values (σ_i) and (B) basis functions (ψ_i) were obtained as a result of the SVD processing for the matrix containing the obtained spectra, including the calibration spectra, as shown in Figures 1 and S1.</u>

According to the above description, the linear combination containing the top 3 components was expected to reproduce the spectra at 90.0% accuracy. Figure S3A-S3C shows the first, second, and third singular vectors corresponding to IMP results (cf. Figure 1A) in the SVD procedure for the 162 obtained spectra. With the first basis function (ψ_1) representing the flip-flapped average spectrum, the first component intensity (λ_1) of the singular vector would correspond to the total amount of substrates in the system. Then, the λ_1 maintained at the invariable level is reasonable. It suggested that the λ_2 and λ_3 components would dominantly contribute to reproducing spectral changes.

Similar results were obtained for AMT (cf. Figure 1C) in Figure S3D-S3F. AMT concentration range (0-1.0 mM) was higher than IMP concentration (0-0.4 mM), indicating that the IMP inhibitory effect (or canceling) on the lipid peroxidation (decreasing TBARS at [TRO]=0) was more potent than AMT. The AMT structure's exocyclic double bond differs from the IMP structure's exocyclic alkylamine. That would cause more rigidity in AMT than in IMP. Comparing Appendixes 4 and 7, the AMT structures in proteins are more expanded than the IMP ones. It analogized that the AMT molecule would be inappropriate for intruding into the perturbed membrane owing to its inability to fit the structural diversity in intermolecular interactions. That would be why AMT was required more than IMP to decrease TBARS in the absence of TRO.

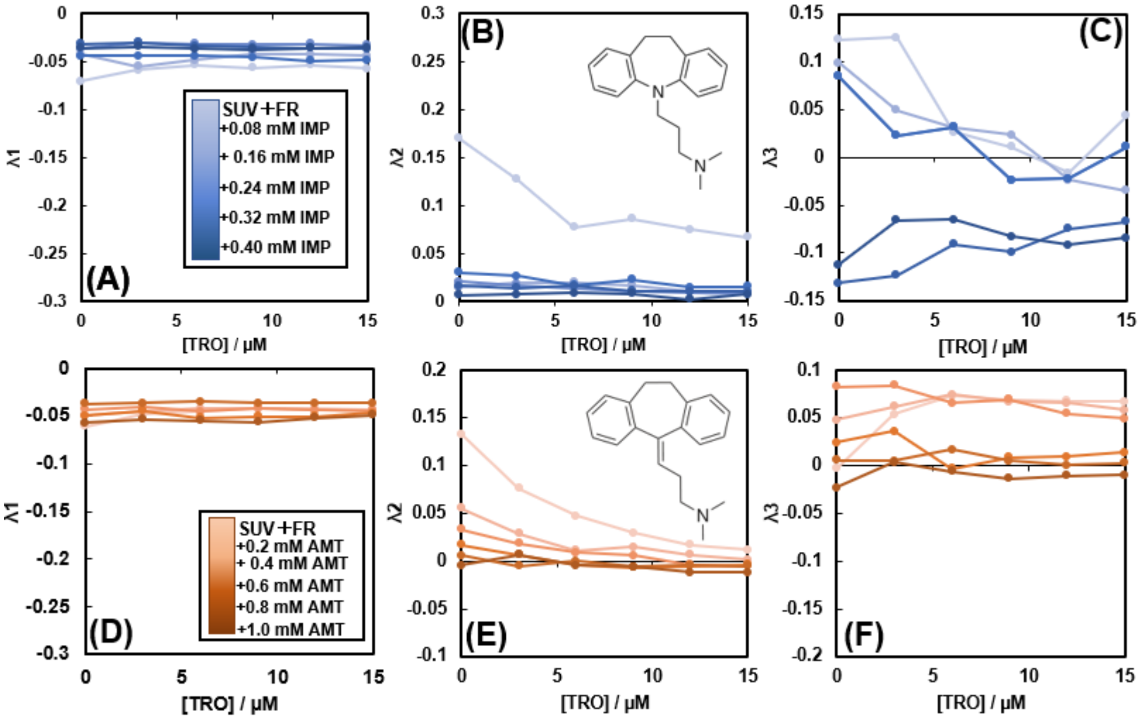

Figure S3. The first, second, and third singular vectors for IMP (A, B, and C) and AMT (D, E, and F) are computed by the SVD processing for the obtained spectra in Figure 1.

Figures S3G-S3I and S3J-S3L show three components of the singular vectors corresponding to the spectra for the DSP and OLZ experiments, cf. Figures 1B and 1D, respectively. DSP's effective concentration range (0-0.5 mM) was comparable to IMP. As the chemical structure of DSP is demethylated from the dimethylamine moiety of IMP, their affinity to the lipid bilayer membrane would be resembled. Comparing Appendixes 4 and 5, the conformations of DSP and IMP under the interaction with proteins are also similar.

Meanwhile, the corresponding activity of OLZ at the concentration range of 0-0.6 μM is more than 80 times the IMP activity at the 0-0.5 mM concentration range. The N-methylpiperazinyl group seems to have poor molecular structural flexibility so that the OLZ structure would be rigid. However, the saturated six-membered ring can take two rigid boats and twenty flexible chair/quasi-chair conformations. As shown in Appendix 1, CLZ showed boat form. Furthermore, the C-N linkage between the N-methylpiperazinyl and the thienobenzodiazepine moieties can switch the anti-/syn- conversion so that OLZ is invested in the high flexibility, which would be comparable to the aliphatic chain. The singular values' first and second components (λ_1 and λ_2) corresponded to the antiparallel and parallel correlation between the peak heights at 455 nm and 530 nm.

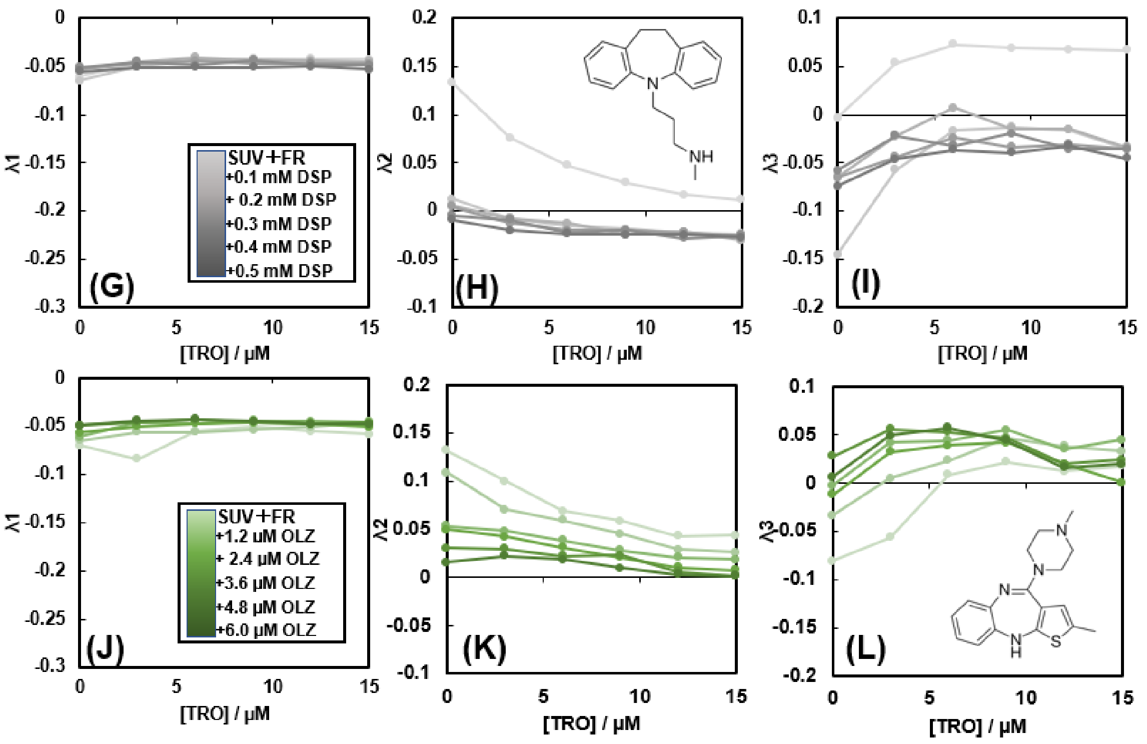

Figure S3. The first, second, and third singular vectors for DSP (G, H, and I) and OLZ (J, K, and L) are computed by the SVD processing for the obtained spectra in Figure 1.

As a result, the contributions of λ_1 and λ_2 are not distinguished, depending on the sample drugs. Therefore, these components were evaluated as the linear combination (ω) of these vector components multiplied by their corresponding singular values.

From the SVD procedures, the maximum range of the TBARS was defined as the distance of the obtained parameters ω_0 and ω_∞, which were optimized by the nonlinear fitting procedures (due to the solver module of Microsoft Excel 2016) onto the regression line. Figure 2 shows a negative logarithm of (ω - ω_∞) divided (normalized) by (ω_0 - ω_∞) as the function of the concentration. Our previous study demonstrated that the local anesthetics LDC and DBC inhibited lipid peroxidation (decreasing TBARS), proportional to their concentration. However, the linear approximations were insufficient to reproduce the inhibitory effects of antidepressants and DHM on lipid peroxidation. Figure 2B shows the upward convex curves. It caused TCAs (IMP and DSP) and SSRIs (PRX, SRT, and FLX) to require comprehensive investigations.

Figure S3M shows the apparent linearity of the concentration would be observed. LDC and DBC (and AMT) might not have enough inhibitory effect on lipid peroxidation. In contrast, TCAs and SSRIs demonstrated upward convex curves at less than 0.5 mM concentrations. Therefore, we attempted that the negative logarithm of (ω - ω_∞) divided by (ω_0 - ω_∞) was represented by the Langmuir-type logistic function, where the evaluation parameters n and K were used.

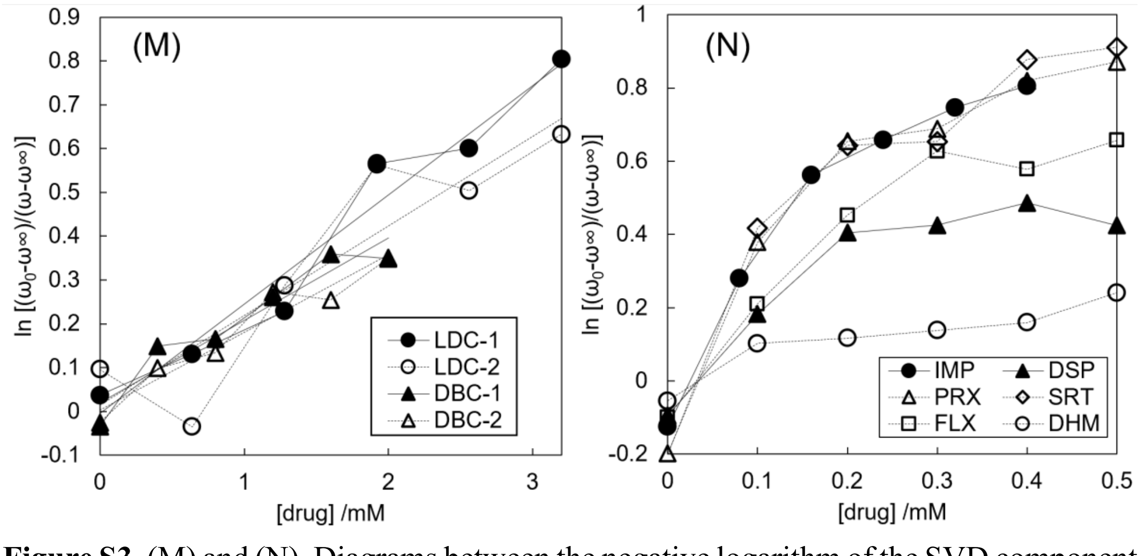

Figure S3. (M) and (N). Diagrams between the negative logarithm of the SVD component score change (ω-ω_∞) relative to the maximum range (ω_0-ω_∞) and the concentrations of 0-3.2 mM LDC, 0-2 mM DBC (M), 0-0.5 mM TCAs and DHM (N), which correspond to Figures 2A and 2B, respectively.

In Figure S3O, the saturation curves were interpreted using the Langmuir-type equation with the saturated intensity n and the association constant K. The sum of squares (SS) was minimized, optimizing the n and K due to the solver module of Microsoft Excel 2016. Satisfied approximations were obtained; the pI50 values resulted in DHM 3.33, IMP 3.52, DSP 3.68, SRT 3.56, FLX 3.44, and PRM 3.60. These temporal values were slightly different from Table 1.

As shown in Figure S5, the local anesthetics and TCAs had almost no scavenging activity for DPPH radicals. This indicated that the inhibitory effects of TCAs and SSRIs on liposomal lipid peroxidation are indirect to reactive oxygen species. Their protective effect on lipid peroxidation seemed to be caused by membranous stabilization. This possibility allowed us to explain the TCAs and SSRIs to show the saturation curves because of the occupied sites on the membranes.

In Figure S5, OLZ has a slight radical scavenging activity. That could influence the spectral change in TBARS generation in the presence of OLZ. Figure S3L could demonstrate characteristic curves of λ3, reflecting any individual peroxide products of lipid peroxidation in the presence of OLZ. The linearity (not an upward convex curve) for OLZ in Figure 2C probably indicates a different interaction of OLZ with the lipid bilayer membrane. LDC and DBC would adsorb on the lipid interface to the liquid phase, but their association constants would not be high, so they cannot be saturated. TCAs and SSRIs also adsorb onto the lipid interface to be saturated.

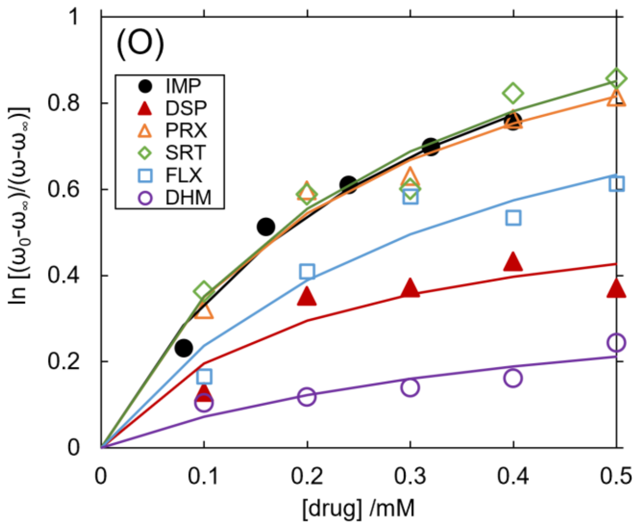

$$\ln\frac{\omega_0-\omega_\infty}{\omega-\omega_\infty}=\frac{n[\mathrm{Drug}]}{K+[\mathrm{Drug}]}$$

Drug	*n*	*K*	*SS*
IMP	1.356	0.303	0.0352
DSP	0.6060	0.210	0.0339
PRX	1.221	0.249	0.0700
SRT	1.320	0.276	0.0756
FLX	1.091	0.360	0.0351
DHM	0.4105	0.469	0.0056
AMT	32.32	43.32	0.297

Figure S3. (O) Diagrams between the negative logarithm of the SVD component score change (ω-ω_∞) relative to the maximum range (ω_0-ω_∞) and the concentrations of 0-0.4/0.5 mM TCAs and DHM, which corresponds to Figure 2(B).

Although OLZ has hydrophobicity similar to or lower than TCAs, its interaction with the lipid membrane might differ from that of TCAs and SSRIs. We considered that OLZ would intrude into the membrane and directly react to peroxidation.

Figure S4A-S4D shows the inhibitory effect of drugs on lipid peroxidation induced by FR in the absence and presence of TRO. In contrast, Figure S4E-S4H illustrates the inhibitory effect of TRO on lipid peroxidation in the presence and absence of drugs. The ω_i is the linear combination of the first and second components derived from the SVD analysis for the observed spectra of the drugs' inhibitory effects on liposomal lipid peroxidation and those of the TRO's inhibitory effect in the absence or presence of medicines. The intensity of the ordinates indicates the inhibitory effect of drugs or TRO on lipid peroxidation. Figures S3N and S3O demonstrate the saturated curves for TCAs and SSRIs, but we simplified that the concentration (abscissa) at ln 2 ≈ 0.693 on the ordinate was defined as the IC_{50} of drugs or TRO from these linear approximations in Figure S4.

The pI_{50} values for TCAs in the presence of TRO and those for TRO in the presence of TCAs might be underestimated a part in Figure 3. As shown in Figures 1C, 2A, and S4H, the dose-response curves for AMT contained scattering, but the estimated pI50 values had a simple dependency on the concentrations of AMT and TRO. As the inhibitory effects of AMT were low, their quantitative accuracy could be expected.

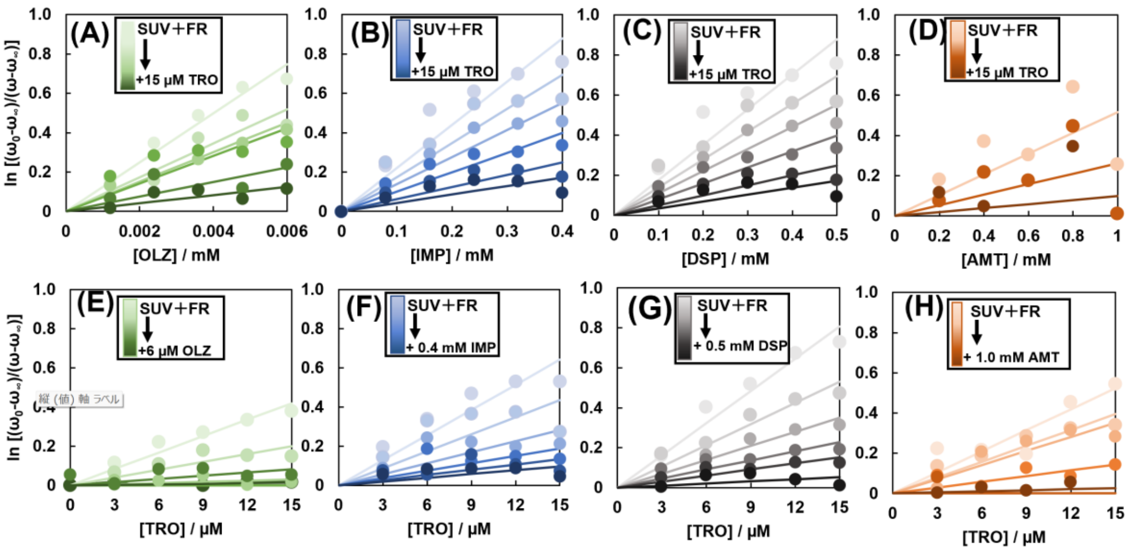

<u>**Figure S4**. Diagrams between the negative logarithm of the SVD component score change (ω-ω_∞) relative to the maximum range (ω_0-ω_∞) and the concentrations of OLZ(A), IMP(B), DSP(C), and AMT(D). Additionally, those to the concentrations of TRO under 0-6 μM OLZ(E), 0-15 μM IMP(F), 0-15 μM DSP(G), 0-15 μM AMT(H).</u>

Quantitative Evaluation of the DPPH Radical Scavenging Activity of TRO in the Presence of Drugs Using the SVD Procedure

Figures 4 and 5 show the inhibitory effect of drugs on the DPPH radical scavenging activity of TRO using ESR spectrometry. As their control experiments, Figure S5 shows the drugs' radical scavenging activity. In Figures S5A-S5E, LDC, DBC, IMP, AMT, and DSP were examined, but their activities were insignificant. Figure S5F shows that OLZ had a radical scavenging activity, as previously described.

The SVD procedure was carried out for the ESR spectra to refine their DPPH radical scavenging activity. Figure S6A shows the singular values, indicating the higher three components with a cumulative contribution of 89.5%. Figure S6B shows the basis functions for the higher three components. The basis function of the third component (gray) was regarded as noise. Then, we recognized that the first and second components were significant.

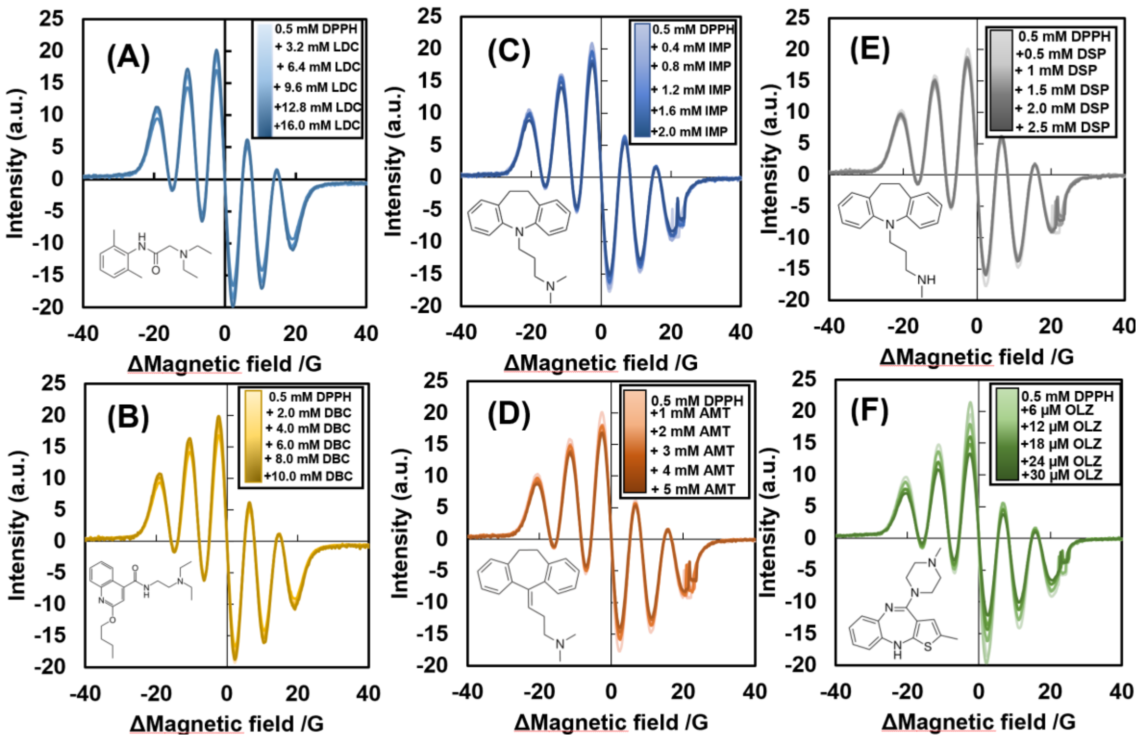

<u>**Figure S5**. The ESR spectra of 0.5 mM DPPH radicals inhibited by 0-16 mM LDC (A), 0-10 mM DBC (B), 0-2.0 mM IMP (C), 0-5.0 mM AMT (D), 0-2.5 mM DSP (E), and 0-30 μM OLZ (F) for 30 min in 71% ethanol and water mixture. The abscissa was aligned to the individual rotatory symmetry point, indicating the magnetic field difference.</u>

Figures S6C and S6D show the component intensity of the first and second singular vectors as a function of TRO. The open circles represent the control (without drugs). In the presence of drugs, the slopes declined compared to the control, indicating the drugs at their own concentrations (they were decided due to the TBARS experiments) interference with the DPPH radical scavenging activity of TRO.

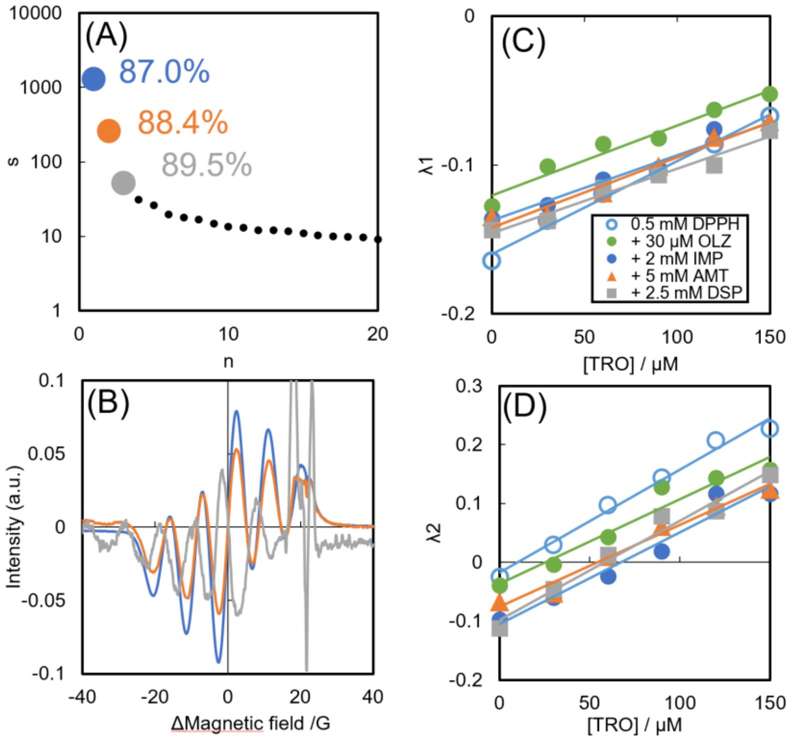

Figure S6. (A) Singular values (σ_i) and (B) basis functions (ψ_i) were obtained as a result of the SVD processing for the matrix containing the obtained spectra, including the calibration spectra, as shown in Figures 4 and S5. The first component ψ_1 (blue) and the second component ψ_2 (amber) include ±2.5, ±10, and ±20 G signals, whereas the third component ψ_3 (gray) seems to be irregular noise. As the singular vectors λ_1 (C) and λ_2 (D) are correlated with each other, we considered the first component as the products of ψ_1, σ_1, and λ_1 would reproduce the observed spectra [60].

The slopes in Figure S7A represented the DPPH radical scavenging activity of TRO, and the addition of drugs decayed that. However, the intercept corresponded to the inhibition due to the drug. Hence, the observed pI_{50} of TRO was insignificantly influenced by the drugs, as shown in Table 1. The slopes in Figure S7B and S7C indicated the drugs' activity. LDC and DBC were incomparable because the inhibition was slightly observed until 10 mM. The IC_{50} activities of IMP, AMT, and DSP were about $10^{-1.6}$ = 1/40 of the TRO's IC_{50}. Meanwhile, OLZ arrived at less than twice the activity of TRO.

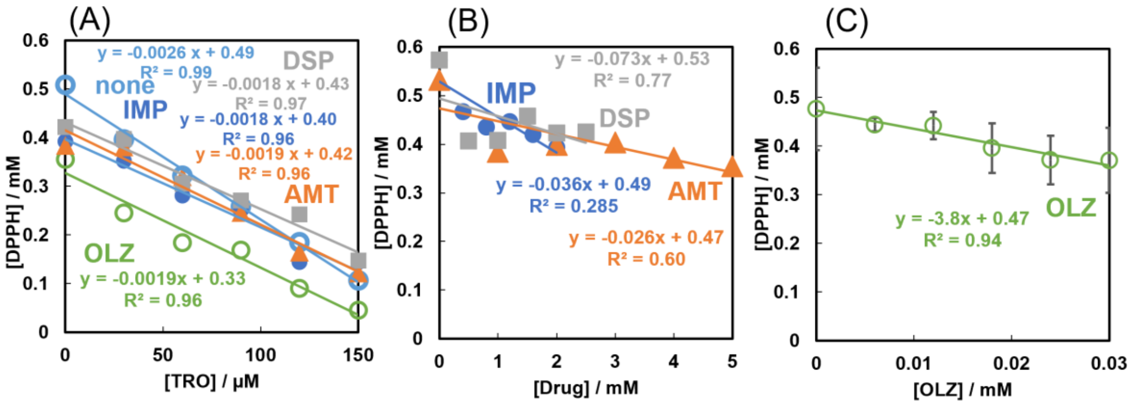

Figure S7. Diagram of DPPH radical consumption to the TRO concentrations in the presence of drugs (A) and those to the TCAs concentrations (B) and OLZ concentrations (C) without TRO.

Quantitative Evaluation of the GLV Radical Scavenging Activity of TRO in the Presence of Drugs Using the SVD Procedure

Figures 4 and S8 show the inhibitory effect of drugs on the GLV radical scavenging activity of TRO using ESR spectrometry. As their control experiments, Figure S8 shows the drugs' radical scavenging activity. In Figures S8B and S8D, DBC and AMT were examined, but their activities were insignificant. Figures S8A, S8C, S8E, and S8F show that LDC, IMP, DSP, and OLZ had radical scavenging activity, as previously described.

The SVD procedure was carried out for the ESR spectra to refine their GLV radical scavenging activity. Figure S9A shows the singular values, indicating the higher three components with a cumulative contribution of 94.2%. Figure S9B shows the basis functions for the higher three components. The basis function of the third component (gray) was regarded as noise. Then, we recognized that the first and second components were significant.

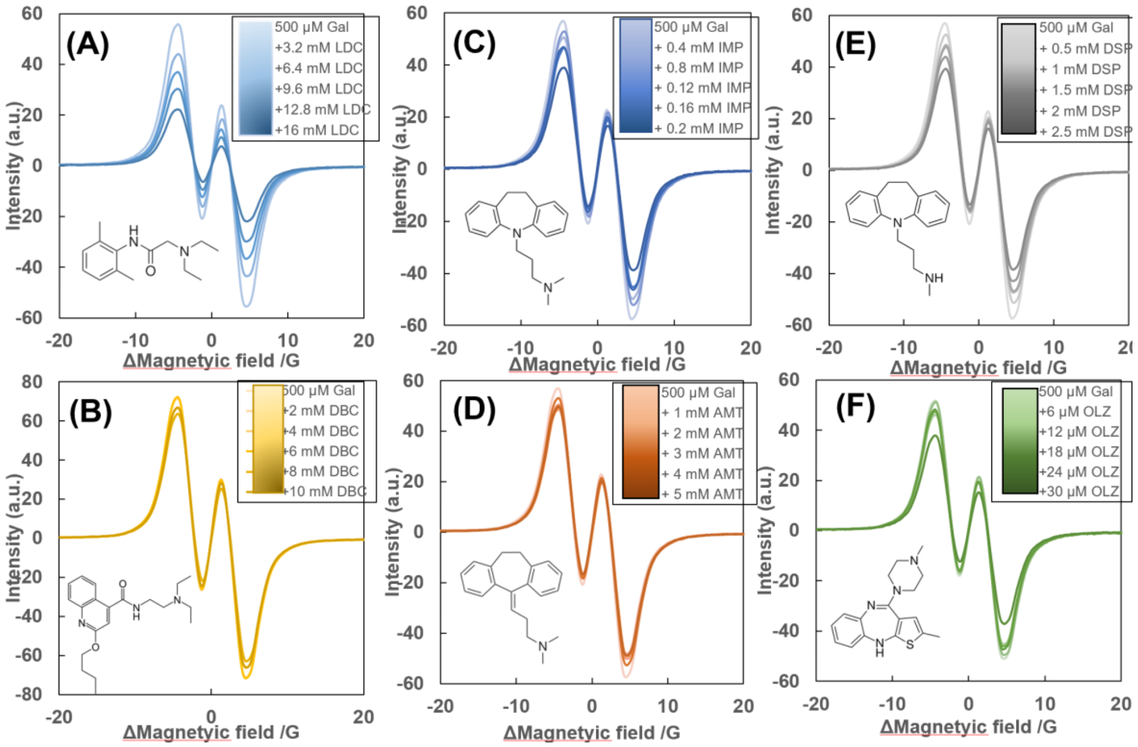

Figure S8. The ESR spectra of 0.5 mM GLV radicals inhibited by 0-16 mM LDC (A), 0-10 mM DBC (B), 0-2.0 mM IMP (C), 0-5.0 mM AMT (E), 0-2.5 mM DSP (E), and 0-30 µM OLZ (F) for 30 min in 1-octanol. The abscissa was aligned to the individual rotatory symmetry point, indicating the magnetic field difference.

Figures S9C-S9F show the component intensity of the first and second singular vectors as a function of TRO. The open squares represent the control (without drugs). In the presence of drugs, the slopes were changed to the control, indicating some drugs at their own concentrations (they were decided due to the TBARS experiments) interference with the GLV radical scavenging activity of TRO.

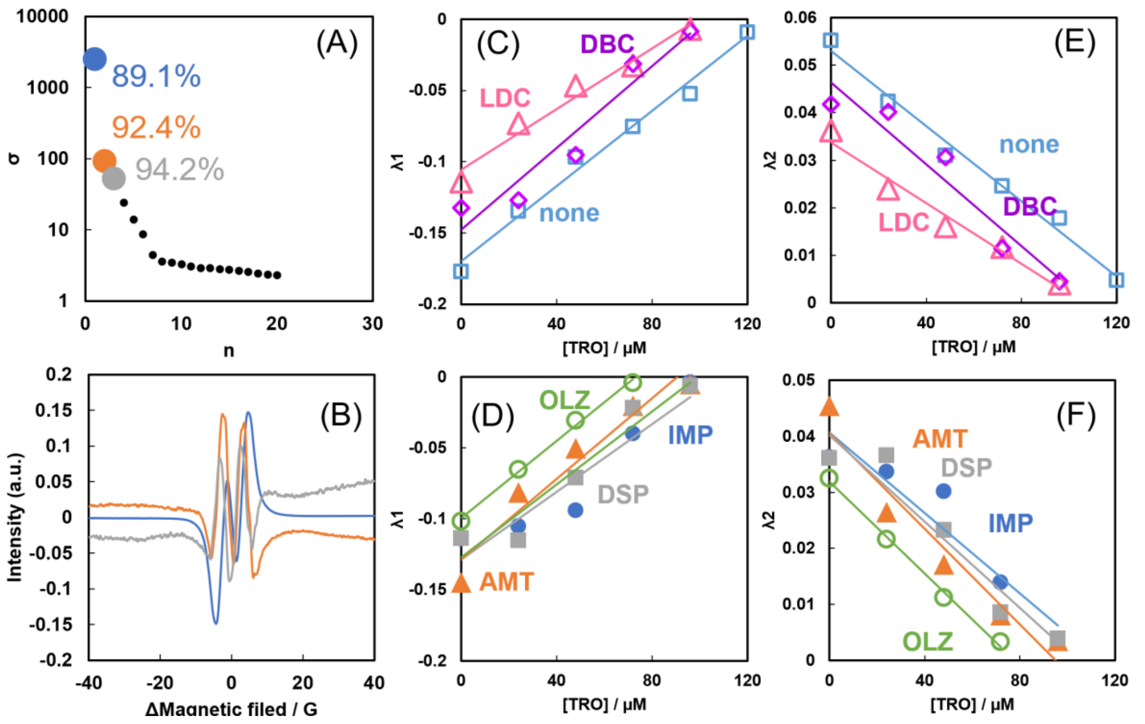

Figure S9. (A) Singular values (σ_i) and (B) basis functions (ψ_i) were obtained as a result of the SVD processing for the matrix containing the obtained spectra, including the calibration spectra, as shown in Figures 5 and S8. The first component ψ_1 (blue) includes ±4.6 G signals, whereas peaks and troughs of the second component ψ_2 (amber) and the third component ψ_3 (gray) seem to differ from the obtained spectra. As the singular vectors λ_1 (C)/(D) and inversed λ_2 (E)/(F) are correlated with each other, we considered the first component as the products of ψ_1, σ_1, and λ_1 would reproduce the observed spectra [60].

The slopes in Figures S10A and S10B represented the GLV radical scavenging activity of TRO, and the addition of drugs decayed that. However, the intercept corresponded to the inhibition due to the drug. Hence, the observed pI_{50} of TRO was insignificantly influenced by the drugs, as shown in Table 1. The slopes in Figures S10C and S10D indicated the drugs' activity. DBC was incomparable. The IC_{50} activities of IMP, AMT, and DSP were less than $10^{-1.7}$ = 1/50 of the TRO's IC_{50}. That of OLZ was similar to the activity of TRO. LDC's activity significantly depended on the concentration, which was more significant than milli-molar.

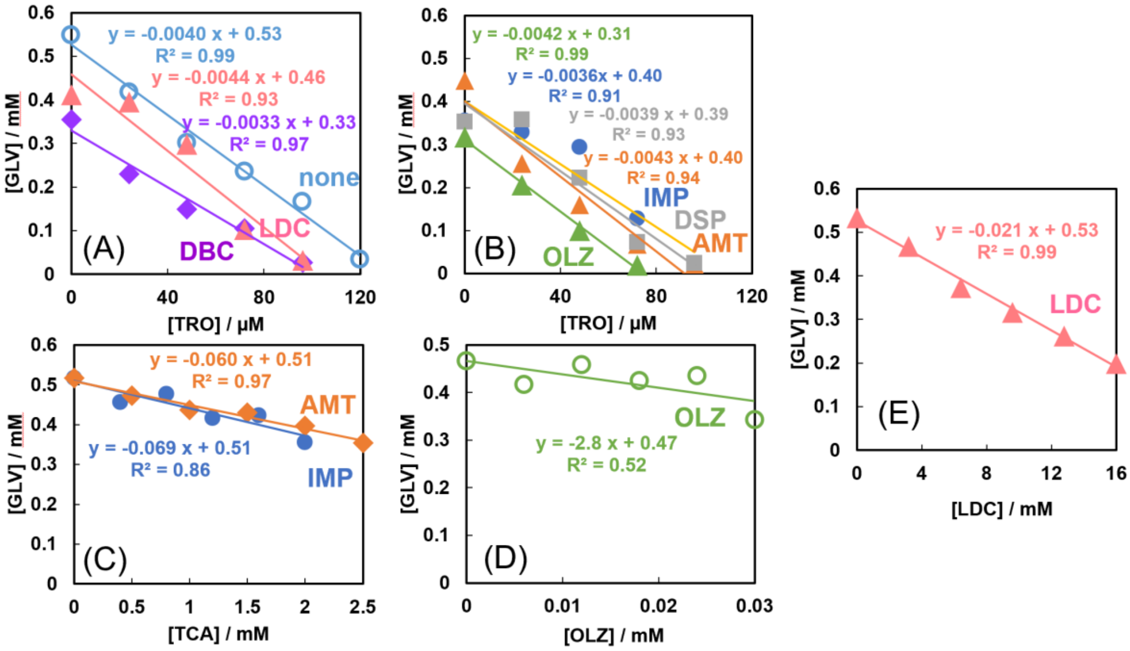

Figure S10. Diagram of GLV radical consumption to the TRO concentrations in the presence of drugs (A)/(B) and those to the TCAs' concentrations (C), OLZ's (D), and LDC's (E) without TRO.

Thermal Analyses for IMP, AMT, DSP, and OLZ

Figure S11 shows the differential scanning calorimetry (DSC) thermograms for TCAs and OLZ. At their melting temperatures, the fusion enthalpy values were 65.859, 47.435, 28.507, and 72.662 J/g.

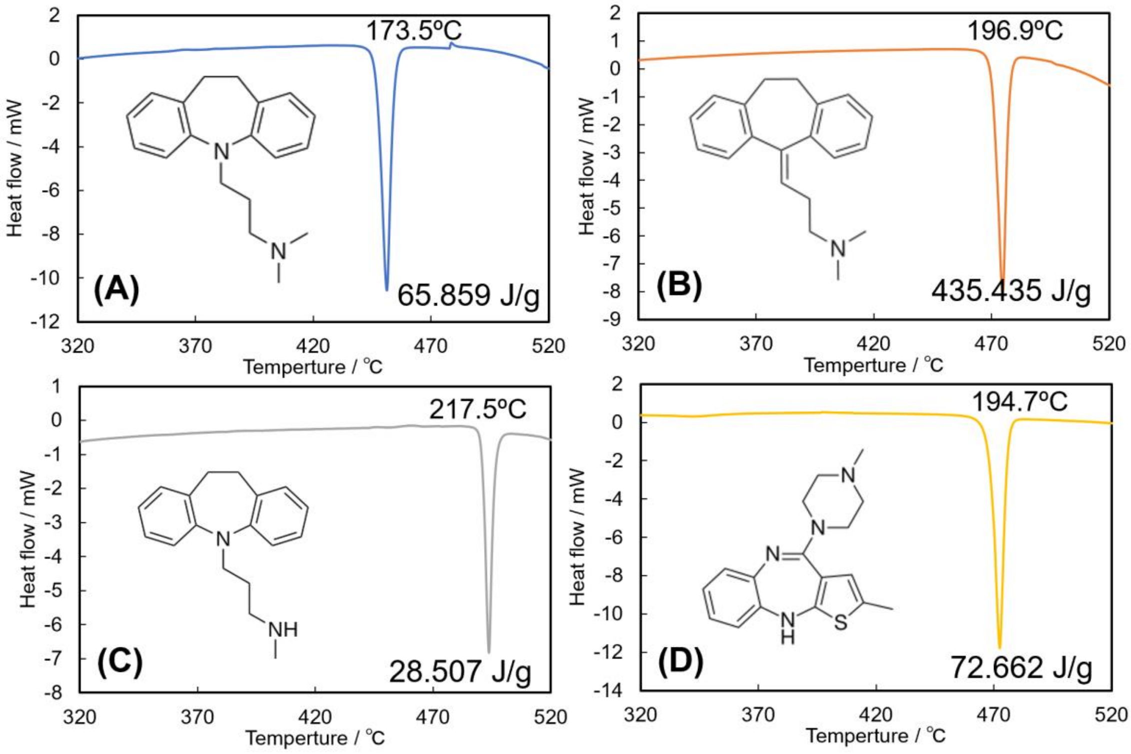

Figure S11. DSC thermograms for IMP (A), AMT (B), DSP (C), and OLZ (D) observed melting temperatures of 173.5, 196.9, 217.5, and 194.7ºC, respectively.

APPENDIX-1
(exposing at the protein/water interface)
CLZ: histamine H4 receptor complex, 8jxv
haloperidol: D2 receptor complex, 6luq
risperidone: D2 receptor complex, 6cm4
risperidone: 5HT2A receptor complex, 6a93

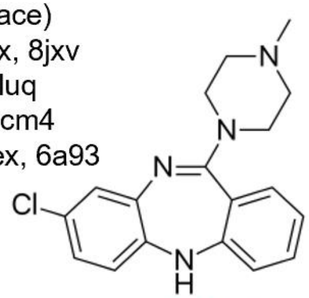

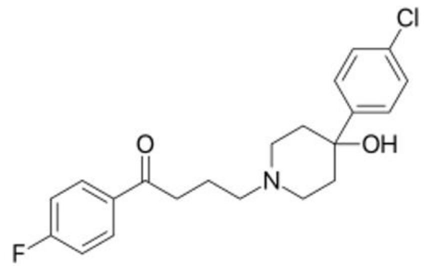

8jxv

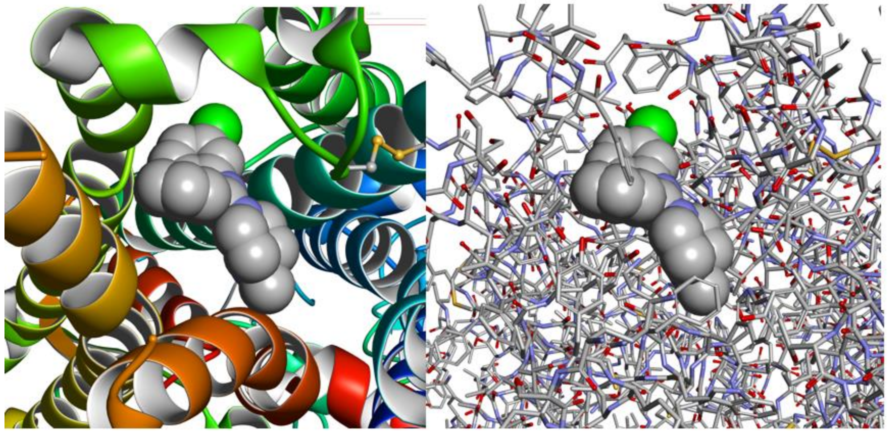

6luq

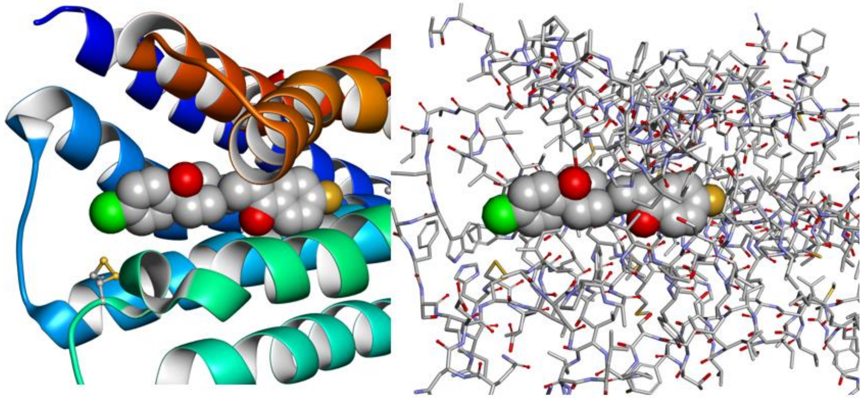

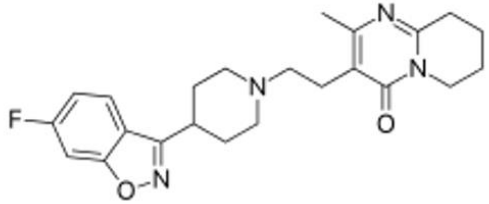

6a93

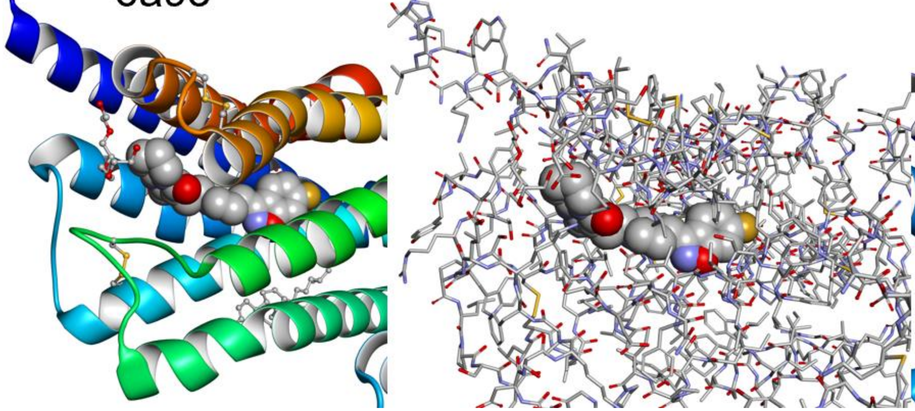

6cm4

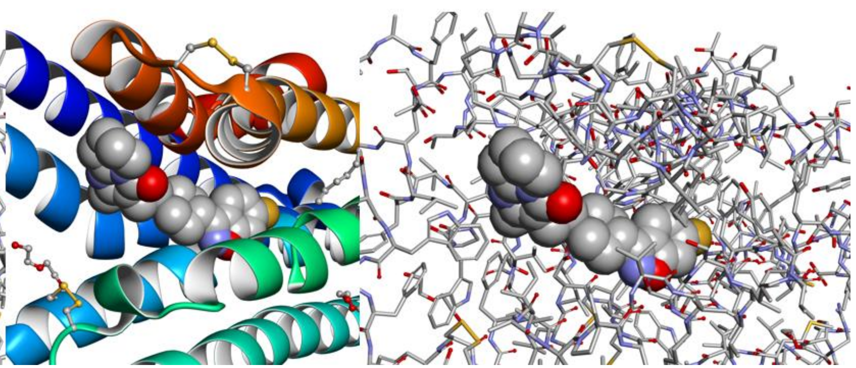

APPENDIX-2
(exposing at the protein/water interface)
diazepam: human serum albumin complex, 2bxf
diazepam: GABA-A receptors, 6x3x
diazepam: GABA-A receptors, 6hup

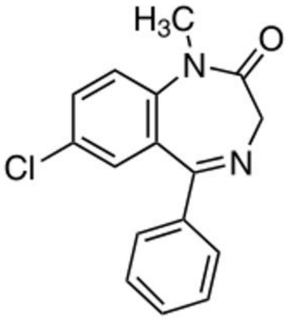

2bxf

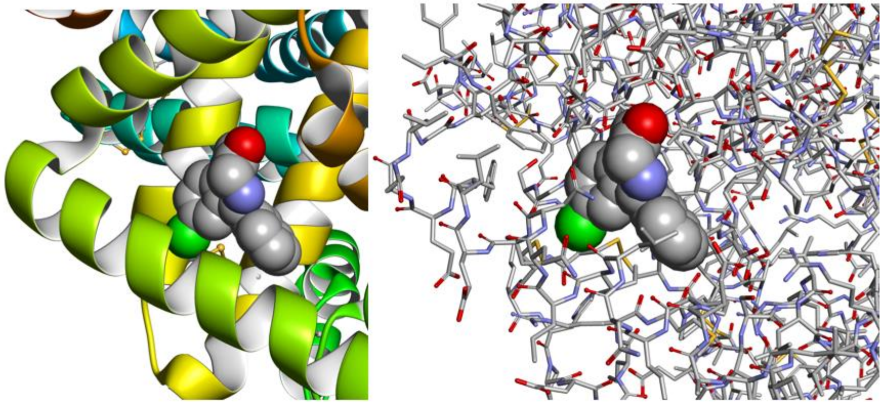

6hup-1 (peripheral loop)

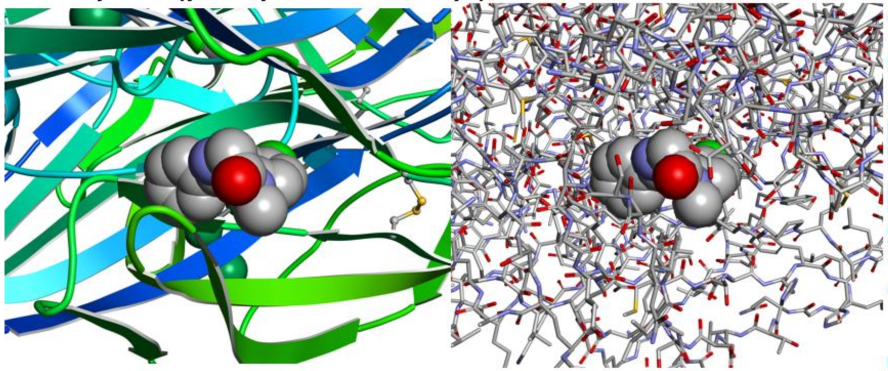

6x3x-1 (peripheral loop)

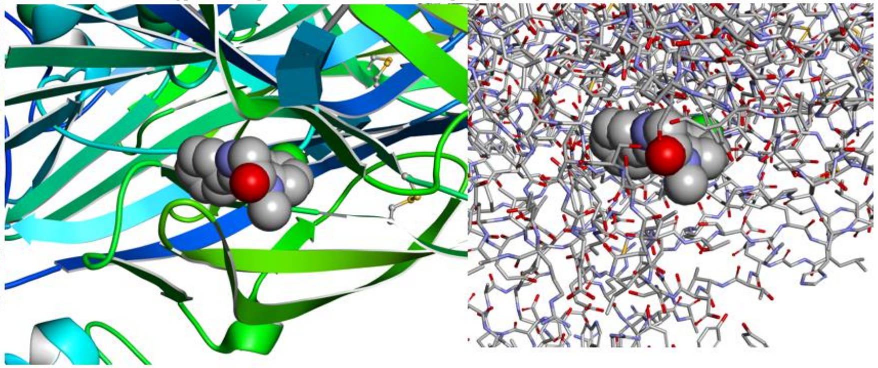

6hup-2 (transmembrane domain)

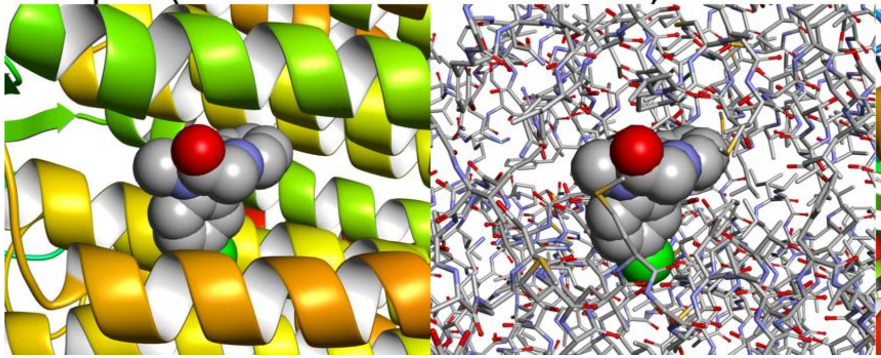

6x3x-2 (transmembrane domain)

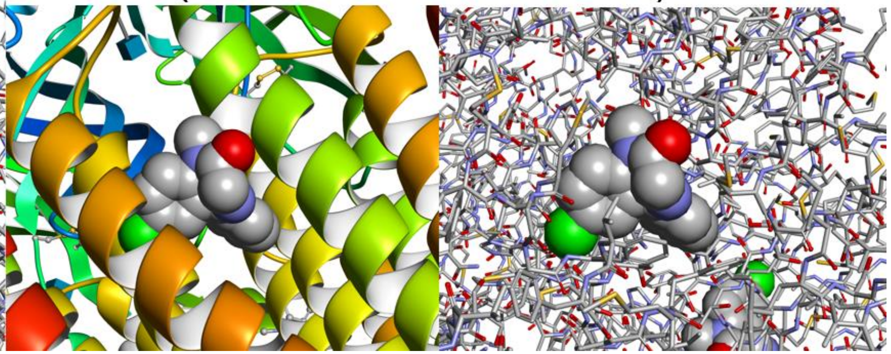

APPENDIX-3
(exposing at the protein/water interface)
Flumazenil: human GABA-A 6x3u
Flumazenil: human GABA-A 6d6t
Flumazenil: human GABA-A 6d6u

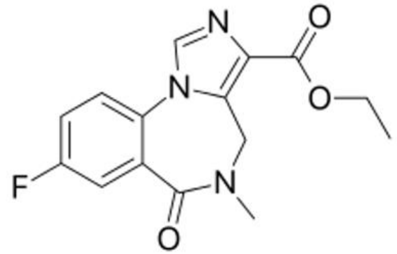

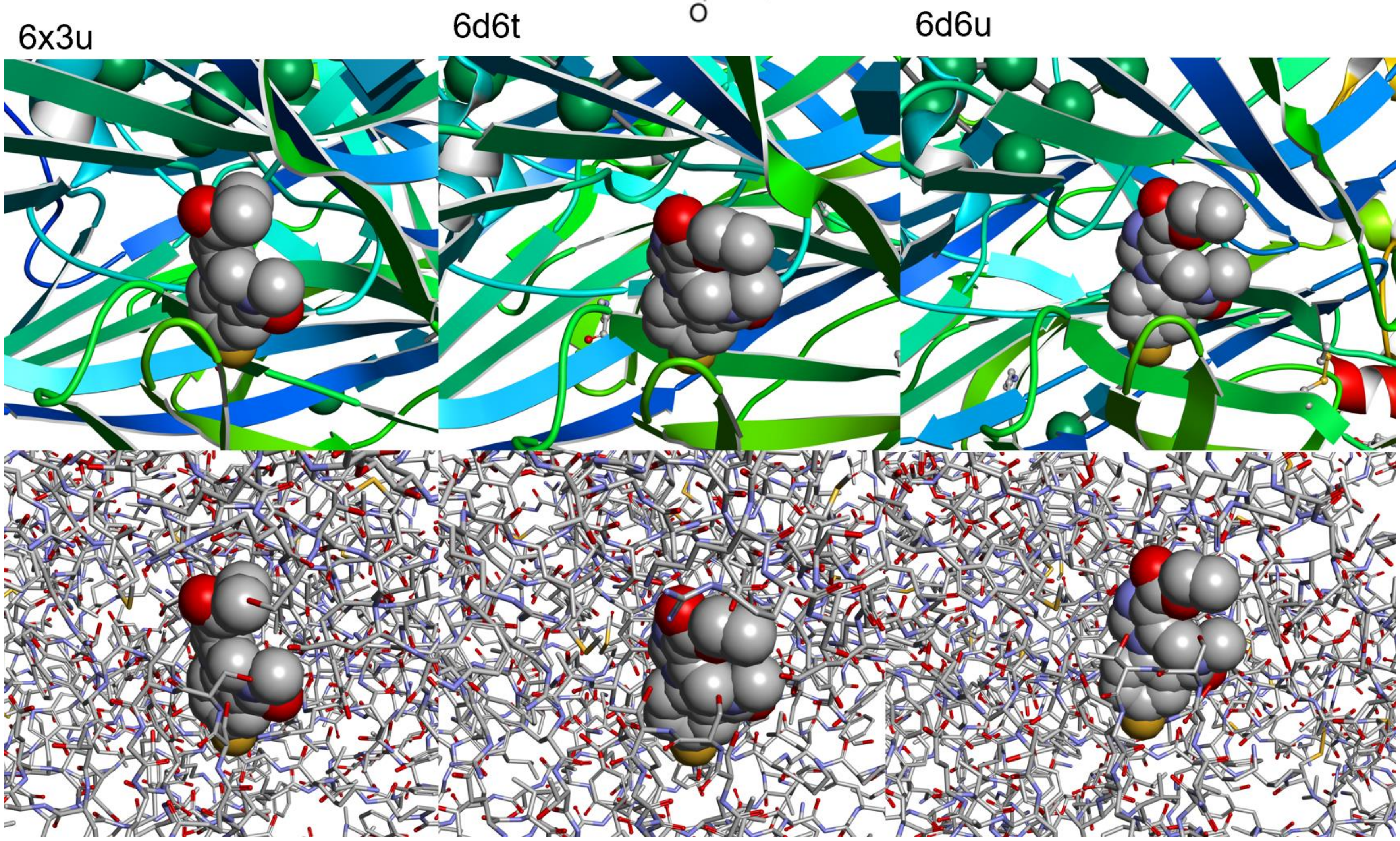

APPENDIX-4
(exposing at the protein/water interface)
IMP: human serotonin transporter, 7lwd
IMP: eubacterium leucine transporter, 2q72
IMP: Ebolavirus glycoprotein, 6g9b

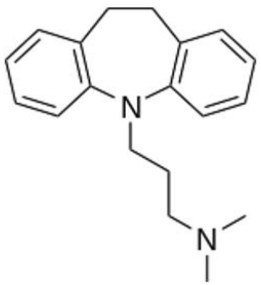

7lwd

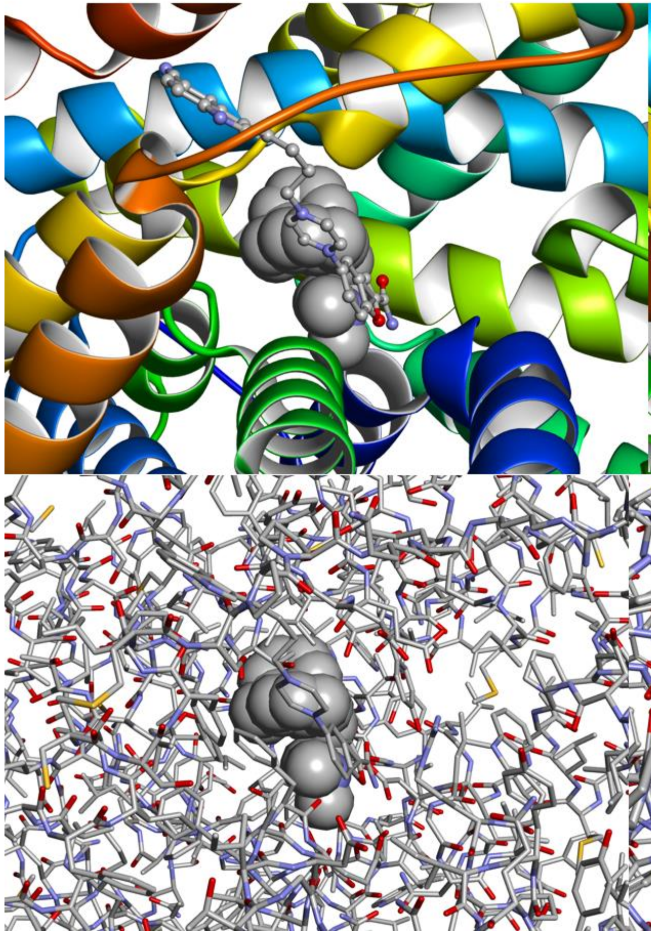

2q72

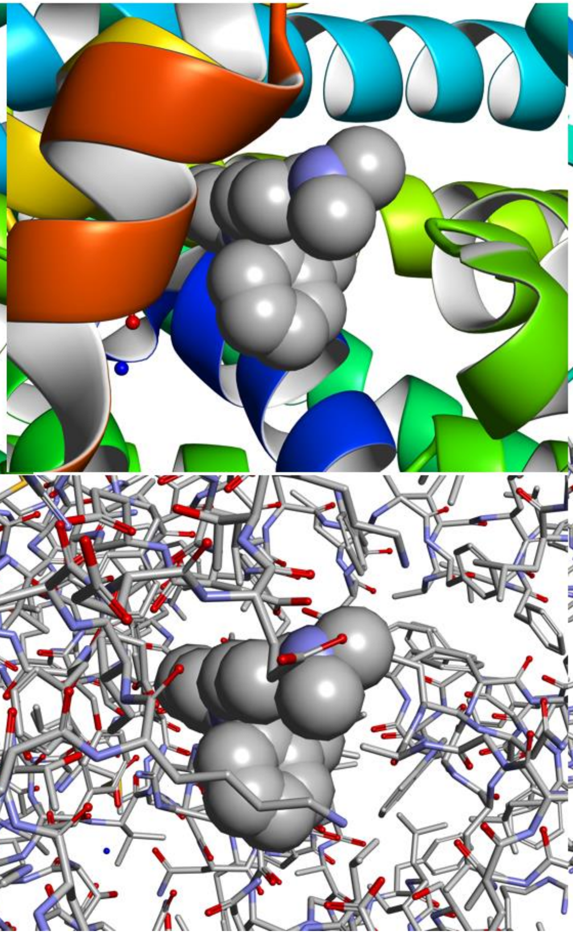

6g9b
(containing 2 molecules)

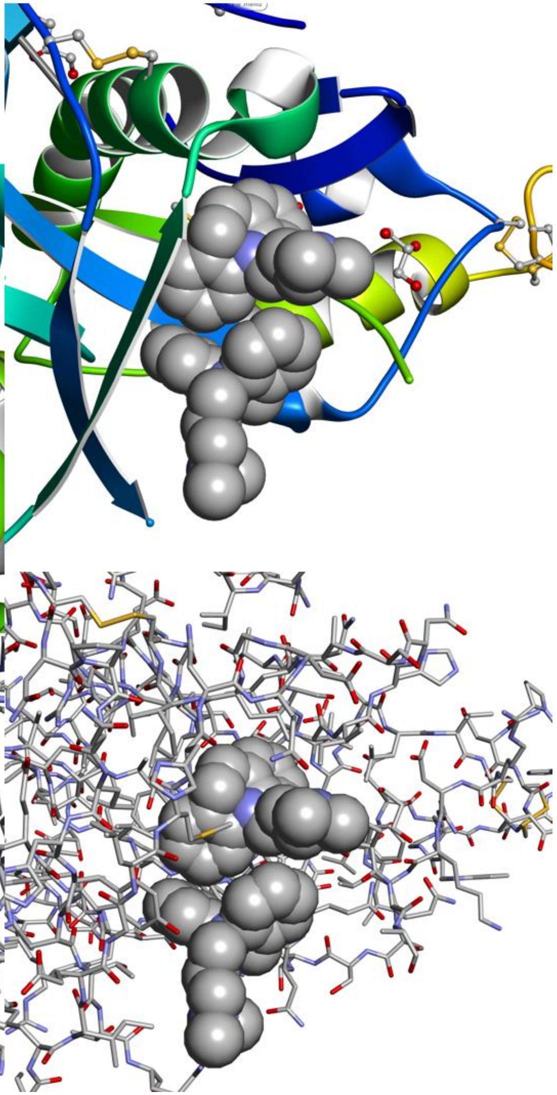

APPENDIX-5 (exposing at the protein/water interface)
DSP: bacterial leucine transporter, 2qb4
DSP: bacterial leucine transporter, 2qju
DSP: β-lactoglobulin mutant complex, 7q19

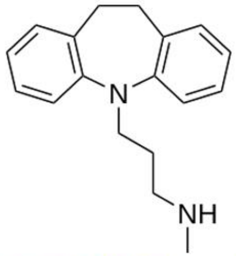

2qb4 2qju 7q19

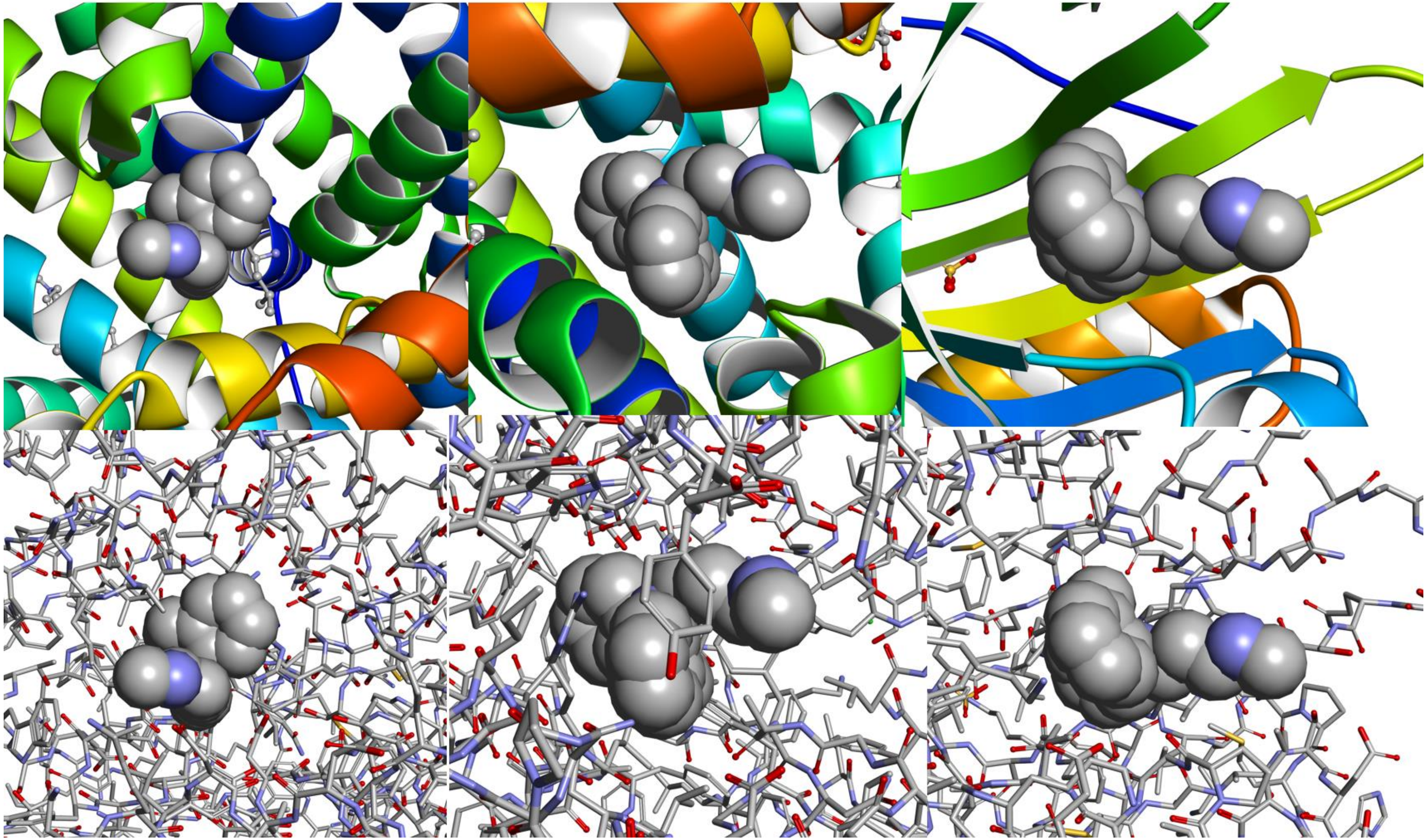

APPENDIX-6 (exposing at the protein/water interface)
CLP: bacterial leucine transporter, 2q6h
CLP: bacterial leucine transporter, 2qei
CLP: Ebolavirus glycoprotein, 6g9i

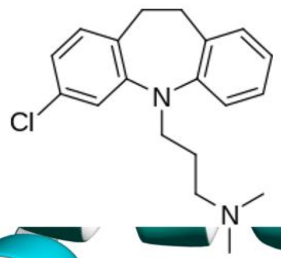

2q6h 2qei 6g9i

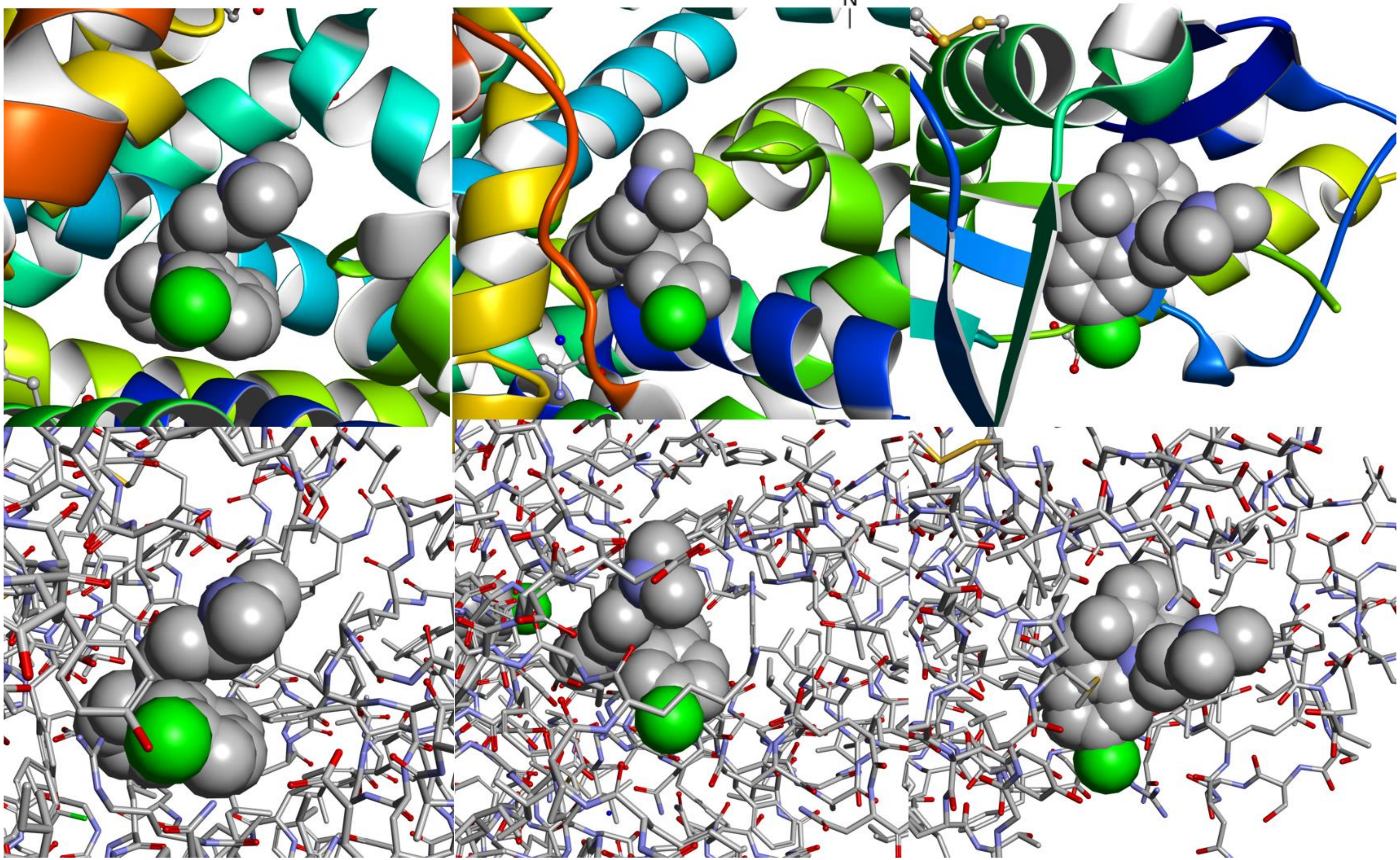

APPENDIX-7 (exposing at the protein/water interface)
AMT: globular protein, 5ha9
AMT: human α1-acid glycoprotein, 3apv
thioridazine: Ebolavirus glycoprotein complex, 6g95

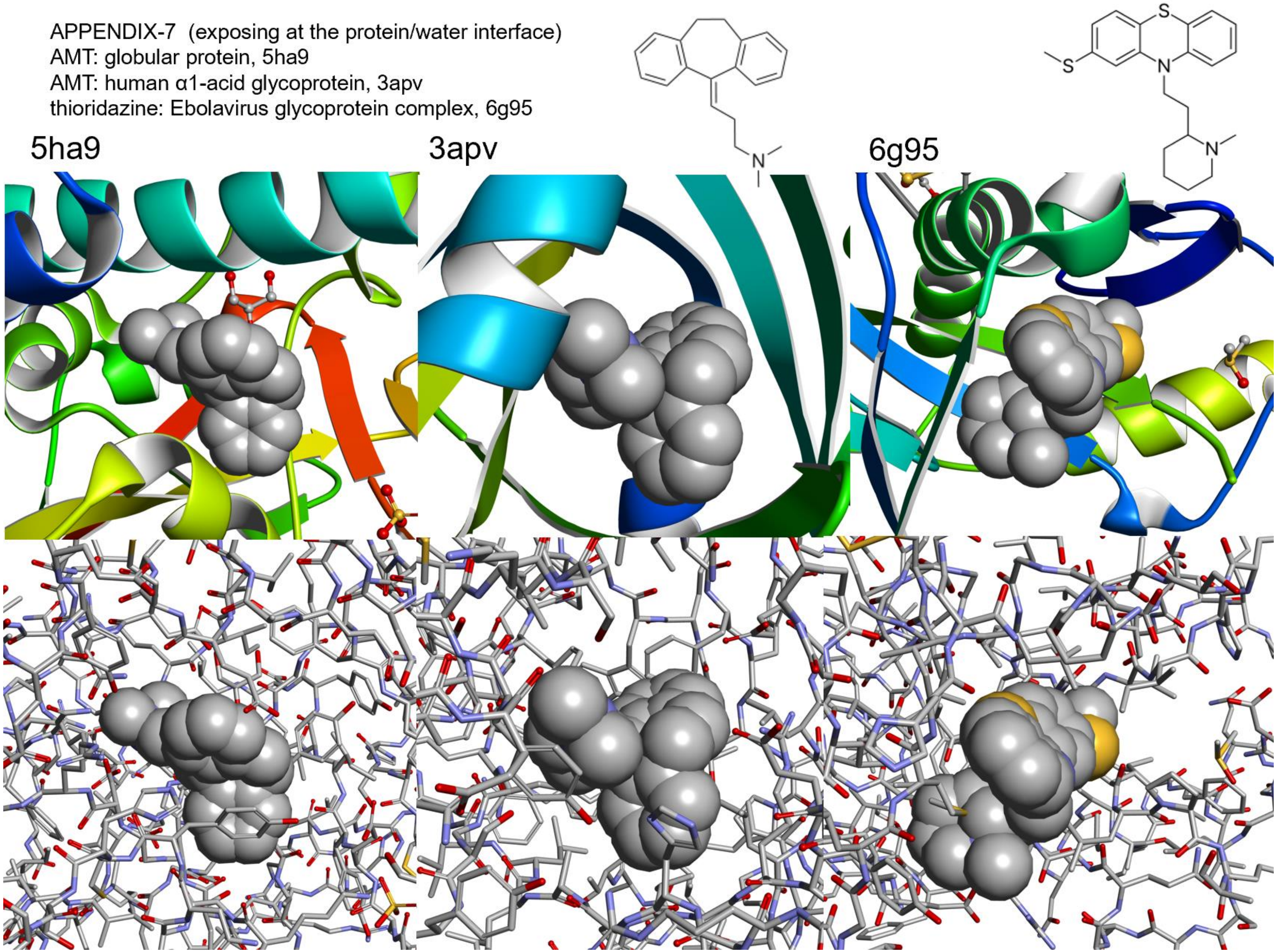

APPENDIX-8 (intruding into the membrane domain)
SRT: human serotonin transporter 7txt
FLX: human serotonin transporter 6awp
PRX: human serotonin transporter 6dzw

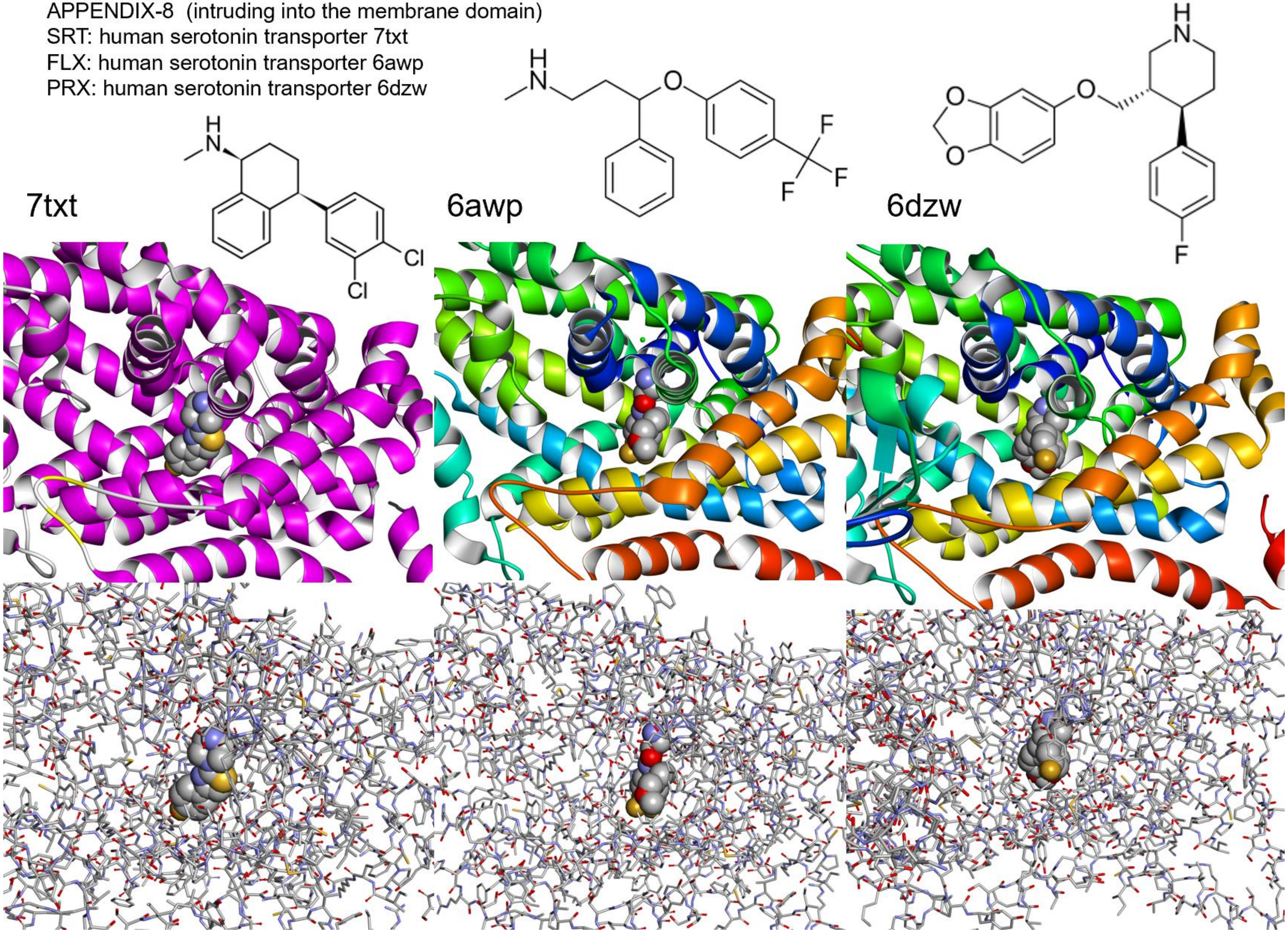

Supplement: Supplementary file 1 — cn4c00702_si_001.pdf [file cn4c00702_si_001.pdf]
